# Two-color coincidence single-molecule pulldown for the specific detection of disease-associated protein aggregates

**DOI:** 10.1126/sciadv.adi7359

**Published:** 2023-11-15

**Authors:** Rebecca S. Saleeb, Craig Leighton, Ji-Eun Lee, Judi O’Shaughnessy, Kiani Jeacock, Alexandre Chappard, Robyn Cumberland, Tianxiao Zhao, Sarah R. Ball, Margaret Sunde, David J. Clarke, Kristin Piché, Jacob A. McPhail, Ariel Louwrier, Rachel Angers, Sonia Gandhi, Patrick Downey, Tilo Kunath, Mathew H. Horrocks

**Affiliations:** ^1^ EaStCHEM School of Chemistry, The University of Edinburgh, Edinburgh EH9 3FJ, UK.; ^2^ IRR Chemistry Hub, Institute for Regeneration and Repair, The University of Edinburgh, Edinburgh EH16 4UU, UK.; ^3^ School of Medical Sciences, Faculty of Medicine and Health, and Sydney Nano, The University of Sydney, Sydney, NSW 2006, Australia.; ^4^ Stressmarq Biosciences Inc., Suite 117-1537 Hillside Ave, Victoria, V8T 2C1 BC, Canada.; ^5^ UCB Biopharma S.P.R.L., Braine l’Alleud, Belgium.; ^6^ The Francis Crick Institute, 1 Midland Road, London NW1 1AT, UK.; ^7^ Department of Clinical and Movement Neurosciences, UCL Queen Square Institute of Neurology, Queen Square, London WC1N 3BG, UK.; ^8^ Aligning Science Across Parkinson’s (ASAP) Collaborative Research Network, Chevy Chase, MD 20815, USA.; ^9^ Centre for Regenerative Medicine, Institute for Stem Cell Research, School of Biological Sciences, The University of Edinburgh, Edinburgh EH16 4UU, UK.

## Abstract

Protein misfolding and aggregation is a characteristic of many neurodegenerative disorders, including Alzheimer’s and Parkinson’s disease. The oligomers generated during aggregation are likely involved in disease pathogenesis and present promising biomarker candidates. However, owing to their small size and low concentration, specific tools to quantify and characterize aggregates in complex biological samples are still lacking. Here, we present single-molecule two-color aggregate pulldown (STAPull), which overcomes this challenge by probing immobilized proteins using orthogonally labeled detection antibodies. By analyzing colocalized signals, we can eliminate monomeric protein and specifically quantify aggregated proteins. Using the aggregation-prone alpha-synuclein protein as a model, we demonstrate that this approach can specifically detect aggregates with a limit of detection of 5 picomolar. Furthermore, we show that STAPull can be used in a range of samples, including human biofluids. STAPull is applicable to protein aggregates from a variety of disorders and will aid in the identification of biomarkers that are crucial in the effort to diagnose these diseases.

## Teaser

A sensitive assay is presented that can detect the protein structures that precede symptom onset in neurodegenerative disease.

## INTRODUCTION

Protein aggregation plays a key role in the pathogenesis of many neurodegenerative disorders, including Parkinson’s disease (PD), and Alzheimer’s disease (AD) (*1*). In PD, alpha-synuclein (α-syn) is incorporated into intracellular inclusions, referred to as Lewy bodies and Lewy neurites, whereas in AD, intracellular neurofibrillary tangles of tau protein, and extracellular amyloid-β (Aβ) plaques, are common neuropathological hallmarks. Increasing evidence suggests that the early-stage oligomeric intermediates that precede the formation of these insoluble inclusions are the cytotoxic species (*2*, *3*). Given their importance in disease pathogenesis, these oligomers are an exciting candidate biomarker of disease progression, with elevated levels of oligomeric α-syn detected in PD patient cerebrospinal fluid (CSF) by enzyme-linked immunosorbent assay (ELISA) (*4*). However, oligomers present as a heterogeneous population, consisting of different sizes, structures, and proteoform compositions (*5*), and the physical characteristics that differentiate disease-associated oligomers from benign forms are largely unknown. This is in part due to ensemble averaging approaches being unable to stratify oligomer populations. To address this, there is a need for advances in technology capable of detecting and characterizing the toxic oligomeric species. Achieving this will have notable implications for patient stratification and the evaluation of clinical trial outcomes.

Protein adsorption onto glass enables direct imaging at the single-molecule level using total internal reflection fluorescence (TIRF) microscopy. This method, however, prevents the use of antibodies for labeling as they are also adsorbed onto the glass surface. We and others have successfully circumvented this hindrance by using nonspecific amyloid dyes to target protein aggregates by single aggregate visualization by enhancement (SAVE) imaging (*6*). While SAVE and related approaches have enabled the detection (*6*) and even the nanoscopic imaging of oligomers in CSF (*7*, *8*), they rely on nonspecific dyes, such as thioflavin-T (ThT), which binds a cross–β structural motif commonly found in amyloid structures.

To overcome this and access the advantages of protein-specific immunodetection, several surface treatment methods have been developed to minimize nonspecific adhesion of biomolecules to the glass surface. When it comes to single-molecule detection, single-molecule pulldown (SiMPull) is the gold-standard surface passivation method (*9*). In this approach, the surface is blocked by covalent modification with polyethylene glycol (PEG) molecules, a fraction of which are biotinylated to enable the specific addition of streptavidin and biotinylated antibody. The immobilized protein target is then immunolabeled and imaged using TIRF microscopy. Je *et al.* (*10*) have demonstrated use of this technique to visualize total α-syn in human brain homogenates; however, higher-order species can only be inferred from the intensity of the fluorescent signal, resulting in an underestimation of the prevalence of oligomers in a sample.

In this work, we modified SiMPull to specifically detect protein aggregates against a background of monomeric protein using two-color coincidence detection (*11*). Our approach, termed single-molecule two-color aggregate pulldown (STAPull), enables detection of picomolar levels of oligomeric α-syn, representing a threefold improvement in sensitivity over single-color detection. Furthermore, unlike ThT-based assays, STAPull was able to distinguish aggregates composed of different amyloid proteins with a high degree of specificity, including early-stage oligomers lacking the extended β sheet structure required for ThT reactivity. Last, we measured aggregates in a range of biological samples, including conditioned media and in life patient CSF, observing substantially higher titers in cases of pathology.

## RESULTS

### STAPull distinguishes oligomeric α-syn with physiologically relevant sensitivity

**Fig. 1.**
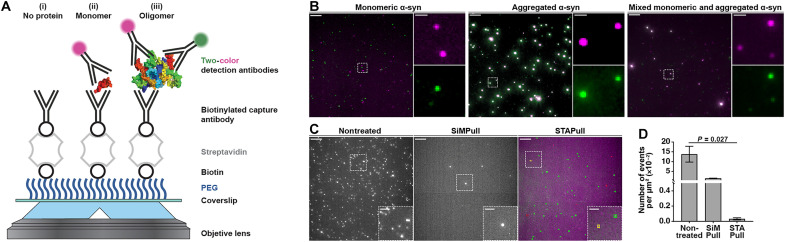
STAPull concept. (**A**) Glass coverslip is silanized and conjugated to PEG, of which 5% is biotinylated, and treated sequentially with streptavidin, biotinylated capture antibody, and the target biomolecule to surface-immobilize. Following incubation with the sample, pulled-down protein is probed using a mixture of AF488- and AF647-labeled monoclonal detection antibody such that (i) no bound protein produces no signal, (ii) monomeric protein produces single-color signal, and (iii) oligomeric protein produces two-color signal via TIRF microscopy. (**B**) Demonstrative composite STAPull images for 45 nM α-syn monomer (left), 5 nM α-syn aggregates (center), or both (right). Monomer is visible as single-color puncta (green or magenta) and oligomers as two-color (white). Single-channel enlargements of the indicated regions shown. Scale bars, 5 μm; crops, 1 μm. (**C**) Representative images of the background signal resulting from adsorption of detection antibody in the absence of target protein to clean nontreated (left), SiMPull (center), or STAPull (right) surfaces. In the latter case, the channel of local maxima are indicated with colored boxes to highlight whether single-channel (green and red) or two-channel (yellow). Scale bars, 5 μm; insets, 2 μm. (**D**) Mean density and SD of detections in (C) (*n* = 3, 16 technical repeats), statistical significance determined using a Kruskal-Wallis test (refer to table S2 for Dunn’s post hoc pairwise means comparison analysis).

To directly visualize α-syn oligomers, they were first immobilized on a PEGylated surface using a biotinylated anti–α-syn capture antibody tethered to the surface via biotin-streptavidin bridging (*9*). Unlike SiMPull, which relies on arbitrary intensity–based filtering to delineate the oligomer population, STAPull uses a 1:1 combination of Alexa Fluor 488 (AF488)–labeled and Alexa Fluor 647 (AF647)–labeled monoclonal detection antibodies (schematized in Fig. 1A). In this approach, the increased epitope availability of higher-order species allows them to be separated from monomeric protein based on the colocalization of two or more orthogonally labeled antibodies. Similar dual-ELISA approaches that use the same capture and detection antibodies have been proposed (*4*); however, we observed that at the single-molecule level, use of a capture antibody targeting a different epitope region to the detection antibodies could improve assay sensitivity (fig. S1).

As recombinant α-syn can self-assemble into fibrils, accelerated by agitated incubation at 37°C, we prepared monomeric and aggregated samples as an in vitro model of pathology. An α-syn–specific STAPull surface was prepared by coating with SYN-CT1 antibody and probing with SYN-CT2 antibody (antibodies provided by UCB Biopharma and detailed in table S1). Application of nanomolar concentrations of monomeric or aggregated protein to the STAPull surface demonstrated increased two-color coincident events in the latter case, indicative of oligomeric species (Fig. 1B). This finding could be replicated using commercially sourced antibodies (fig. S2) and, crucially, the morphology and concentration of the structures were unaltered by antibody binding, as confirmed by assessing single-molecule Förster resonance energy transfer (FRET) in the presence and absence of antibodies as previously published (*12*) (fig. S3). On the basis of the ratio of coincident to total detections, we observed that aggregated α-syn was composed of ~44.5% oligomeric species, while “pure” monomer contained ~6.8% oligomer. Combining these two solutions at a molar ratio of 1:9 produced a solution containing 11.6% aggregated protein as would be theoretically anticipated based on the above stoichiometry.

The primary aim of surface passivation is to block nonspecific protein adsorption; however, perfect passivation is unfeasible and low-level background signal persists (*13*). STAPull minimizes this residual background by rejecting single-channel nonspecific events alongside monomeric signal. To demonstrate this, we compared the background signal produced by exposing SiMPull, STAPull, or nontreated surfaces to 1 nM labeled antibody in the absence of target protein (Fig. 1, C and D). SiMPull surface passivation provides a ~10-fold reduction in nonspecific binding compared with a nontreated surface (mean ± SD: 0.1377 ± 0.0403 events/μm^2^ versus 0.0140 ± 0.0012 events/μm^2^). This can be enhanced by a factor of ~40 when only coincident events are measured using STAPull (0.0003 ± 0.0001 events/μm^2^) (Fig. 1D).

**Fig. 2.**
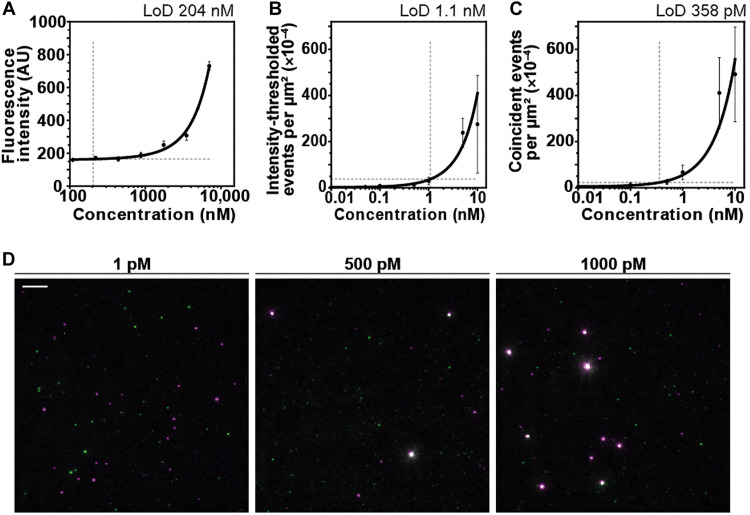
Improved limit of detection using STAPull. (**A**) ThT fluorescence spectroscopy of α-syn aggregates as a function of monomer-equivalent α-syn concentration. (**B**) Single-molecule α-syn aggregate density measured by intensity-thresholded SiMPull or (**C**) STAPull as a function of monomer-equivalent α-syn concentration. Dashed lines indicate the LoD, and the corresponding concentration is determined via a two-order polynomial fit (black line). Data are means of *n* = 3 (each averaged over 64 technical repeats) and SD shown. (**D**) Representative images of STAPull with α-syn aggregate concentration below (1 pM), near (500 pM), and above (1000 pM) its LoD. Scale bar, 5 μm.

To determine whether this reduction in background signal led to improved detection sensitivity, we measured a dilution series of aggregated α-syn and determined the lowest concentration of protein that could be reliably detected (*14*). STAPull produced a limit of detection (LoD) of 22.6 × 10^−4^ events/μm^2^, or 358 pM monomer equivalent α-syn, which was a 3-fold improvement in sensitivity compared to SiMPull and near 600-fold improvement on ThT fluorimetry (Fig. 2, A to C). To approximate the oligomer concentration this equates to, we used the average intensity of single-channel events in a pure monomer sample to estimate the average number of monomer units per aggregate as 30.5 monomeric units. Though such an approach cannot determine absolute stoichiometry because of challenges of epitope accessibility and variable dye molecules per antibody, we confirmed that there is a strong positive correlation between oligomer size and antibody intensity (fig. S4), and our resulting estimate of oligomer size aligns well with previously validated oligomer sizes (*15*). When accounting for both oligomer size and the 44.5% oligomer load determined above, STAPull has an approximate LoD of 5 pM oligomer concentration. This brings STAPull sensitivity into a physiological range, with the oligomer content of PD patient CSF determined by ELISA to be ~10 pM (*16*, *17*). We attribute this improvement over intensity-thresholded SiMPull to both STAPull’s increased signal-to-background ratio and its independence from sample- or technique-derived intensity inconsistencies.

### Specific detection of protein aggregates using STAPull

**Fig. 3.**
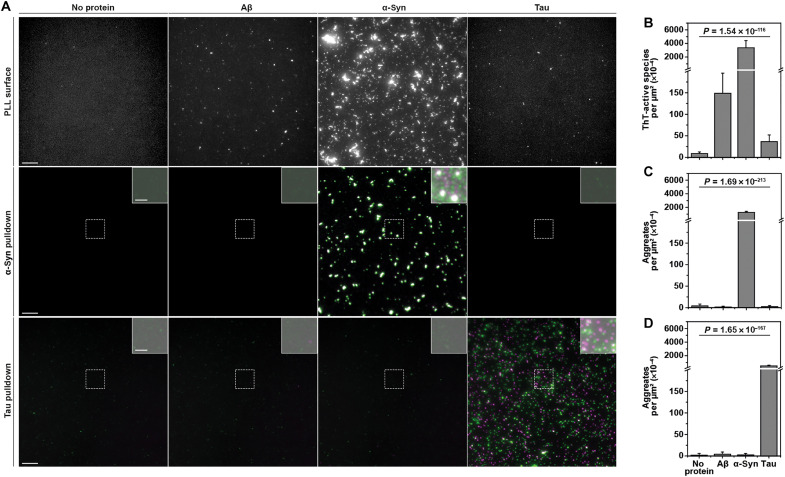
STAPull provides protein-specific aggregate detection. (**A**) Representative TIRF microscopy images of different surface preparations exposed to aggregated Aβ, α-syn, tau, or no protein as indicated. Protein probed with (top row) ThT by SAVE imaging on a PLL-coated surface, (middle row) two-color antibodies against α-syn on an α-syn–specific STAPull surface, or (bottom row) two-color antibodies against tau on a tau-specific STAPull surface. Enlarged insets of indicated regions are at high contrast with 0.5 gamma adjustment to visualize any low-level signal. Scale bars, 5 μm; inset, 2 μm. (**B**) Mean density of ThT-reactive species detected on the PLL surface, (**C**) mean density of two-color species on the α-syn STAPull surface, and (**D**) the tau surface. A split *y* axis is used to visualize low-level variation [scaled 0 to 200 and 250 to 7000 counts per square micrometer (×10^−4^) before and after break, respectively]. SD of 64 technical repeats shown with statistical significance determined by one-way analysis of variance (ANOVA) analysis (refer to table S2 for details of post hoc Tukey pairwise means comparisons).

SAVE imaging can achieve similar detection sensitivity to STAPull, but its application is limited by an inability to determine protein identity and thus disease type. Conversely, as an epitope-mediated assay, STAPull has the capacity to differentiate the underlying proteinopathy. To verify this, we prepared three different surface types: (i) a poly-l-lysine (PLL) coating to facilitate total protein adsorption for SAVE-based ThT detection, (ii) a STAPull surface with α-syn–specific capture and detection antibodies, and (iii) a STAPull surface with tau-specific capture and detection antibodies. Upon exposure of each surface to aggregates of either Aβ, α-syn, tau, or buffer alone, only the STAPull surfaces exclusively detect the target protein for which they were designed (Fig. 3A). ThT detection, on the other hand, demonstrates nonspecific signal elevation compared to the protein-free control in response to all three amyloid proteins. Notably, the densities of α-syn and tau observed on PLL (3362.38 × 10^−4^ and 36.44 × 10^−4^ events/μm^2^, respectively) are broadly similar, or elevated, on STAPull surfaces (1251.80 × 10^−4^ and 488.95 × 10^−4^ events/μm^2^), despite applying a 50-fold lower concentration to the latter. This highlights the importance of the capture antibody for protein enrichment on the surface and the suitability of STAPull for probing rare oligomeric species in patient biofluids.

Though it would be plausible to create specificity for ThT detection via the capture antibody alone, we and others (*13*) have observed that PEG passivation is unable to completely resist protein adsorption to the surface, necessitating antibody detection to achieve specificity (fig. S5). Furthermore, whereas STAPull can theoretically detect dimeric species, ThT is only able to bind amyloid structures containing extended β sheet structure (*18*).

### STAPull detection of kinetically stable oligomers

We observed that upon assaying different time points of α-syn exposed to conditions favoring its aggregation, it was possible to identify oligomers with STAPull earlier than with ThT detection (fig. S6). As early oligomeric structures are increasingly believed to be the neurotoxic species driving synucleinopathy (*2*), and are most likely present at the earliest stages of disease, a means to detect them would have notable implications for early diagnosis, drug discovery, and the evaluation of clinical trial outcomes. We therefore sought to assess the suitability of STAPull for the detection of these species using kinetically stable oligomers.

**Fig. 4.**
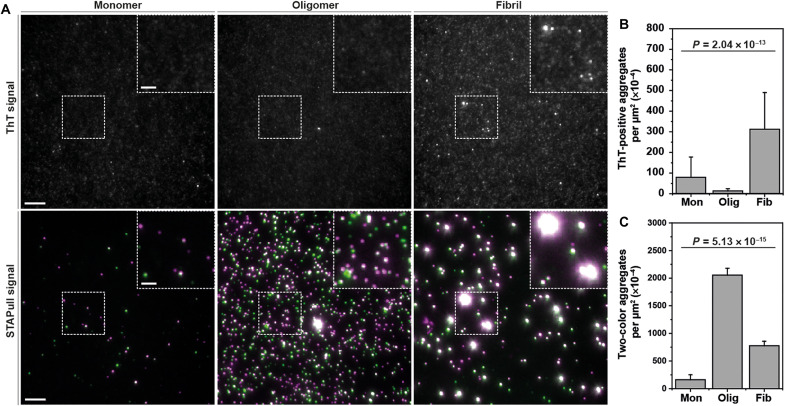
STAPull detects ThT-negative α-syn oligomers. (**A**) Representative SiMPull images of commercially sourced human recombinant α-syn in the monomeric, kinetically stable oligomeric, or preformed fibrillar form as indicated. The same field of view is presented with ThT detection (top) or STAPull two-color coincidence (bottom), with the boxed region magnified in inset. Scale bar, 5 μm in full field and 2 μm in inset. (**B**) Quantification of the mean number of ThT puncta or (**C**) STAPull coincident events per square micrometer for the dataset represented in (A). Error bars indicate SD of 25 technical repeats, and statistical significance was determined using a Kruskal-Wallis test (refer to table S2 for Dunn’s post hoc pairwise means comparison analysis).

Kinetically stable oligomers have previously been characterized as soluble globular α-syn structures with toxic properties demonstrated in vitro (*19*). They appear to have a β sheet structure intermediate between that of the monomer and the fully structured amyloid fibril (*19*). We used two-color coincidence detection and a ThT post-stain to concurrently assay commercially obtained α-syn monomer, kinetically stable oligomers, and preformed fibrils using both STAPull and SAVE imaging. The aggregate species were structurally validated by transmission electron microscopy (fig. S7) to confirm globular and fibrillar structures, respectively. With SAVE imaging, we observed a significant elevation in ThT puncta for the fibrillar species as compared to monomer (mean per square micrometer ± SD: 312.38 × 10^−4^ ± 177.80 × 10^−4^ and 79.24 × 10^−4^ ± 98.21 × 10^−4^, respectively), and these correlated with coincident STAPull detections (Fig. 4, A and B). However, no increase was evident for the oligomer sample (13.57 × 10^−4^ ± 10.46 × 10^−4^), indicating a population of toxic oligomeric species that cannot be detected by ThT-based assays. In contrast, STAPull demonstrated a significantly increased prevalence of coincident detections for both oligomers and fibrils (mean per square micrometer ± SD: 2055.54 × 10^−4^ ± 123.44 × 10^−4^ and 775.37 × 10^−4^ ± 83.30 × 10^−4^, respectively) as compared to monomer (160.58 × 10^−4^ ± 90.53 × 10^−4^), confirming the ability of STAPull to detect these early oligomeric species (Fig. 4C).

### STAPull detection of endogenous protein aggregates

The data presented thus far have demonstrated the suitability of STAPull for the specific detection of early α-syn oligomers generated in vitro. Evidence suggests these species are driving neurotoxicity (*2*), but their detection in biological samples is made challenging because of the complex milieu of biomolecules that may impede immunocapture, nonspecifically bind antibodies, or elevate baseline signal through autofluorescence. Therefore, to determine the suitability of the technique for probing α-syn in these samples, we first used culture medium exposed to human midbrain dopaminergic (mDA) neurons, the primary cell type lost in PD. This “conditioned media” acquires secreted biomolecules and cellular cargo from the cultured cells, thus resembling extracellular fluid and offering a useful in vitro model.

**Fig. 5.**
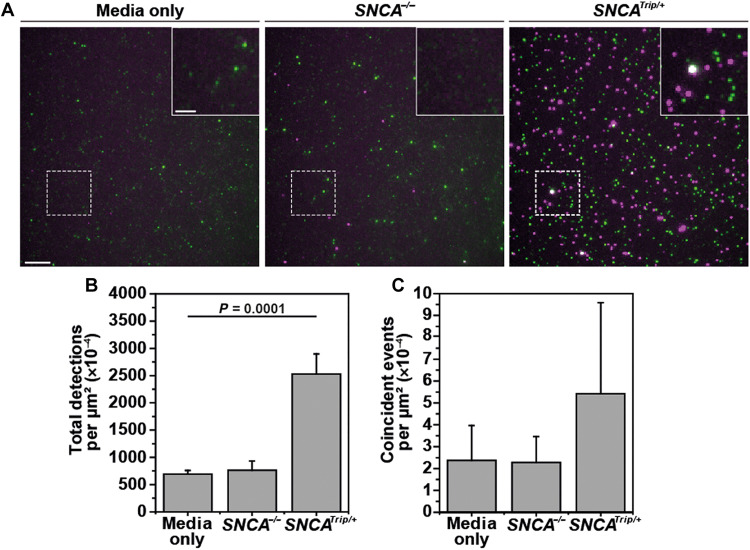
STAPull detects elevated α-syn in iPSC-derived mDA-conditioned media. (**A**) Representative STAPull images of 3-day conditioned culture medium in the absence of cells (left), and in the presence of human iPSC-derived mDA neurons that harbor either a *SNCA* knockout (*SNCA^−/−^*, center) or triplication of the *SNCA* locus (*SNCA^Trip/+^*, right). Monomeric α-syn appear as single-color puncta (green or magenta), and oligomeric appears as two-color puncta (white). Region indicated is magnified in inset. Scale bar, 5 μm; inset, 2 μm. (**B**) Mean density of all detected species (monomeric and oligomeric) and (**C**) coincident-only species, for dataset presented in (A). Error bars show SD (*n* = 3, each with 64 technical repeats) and statistical significance determined by one-way ANOVA analysis (refer to table S2 for details of post hoc Tukey pairwise means comparisons).

mDA neurons were differentiated from induced pluripotent stem cells (iPSCs) derived from a patient with PD harboring a triplication of the α-syn–encoding *SNCA* gene (*SNCA^Trip/+^*) or its CRISPR-engineered knockout (*SNCA^−/−^*). This mutation is known to cause autosomal dominant, early-onset PD due to increased α-syn burden (*20*, *21*) and iPSC-derived neurons exhibit elevated levels of α-syn aggregates (*22*). By using STAPull to assay 3-day conditioned media, we observed a significantly higher total α-syn titer in the *SNCA^Trip/+^* medium than that of medium from *SNCA^−/−^* neurons (mean per square micrometer ± SD: 2528 × 10^−4^ ± 367 × 10^−4^ and 762 × 10^−4^ ± 170 × 10^−4^, respectively). The knockout cells showed no significant elevation above the baseline detections observed in cell-free medium (692 × 10^−4^ ± 66 × 10^−4^) (Fig. 5, A and B). In addition, the number of α-syn aggregates present, indicated by the number of events with coincident signal, also trended upwards in the case of *SNCA^Trip/+^* alone (Fig. 5C), demonstrating that STAPull is sensitive to differences in α-syn oligomer burden in a complex sample.

**Fig. 6.**
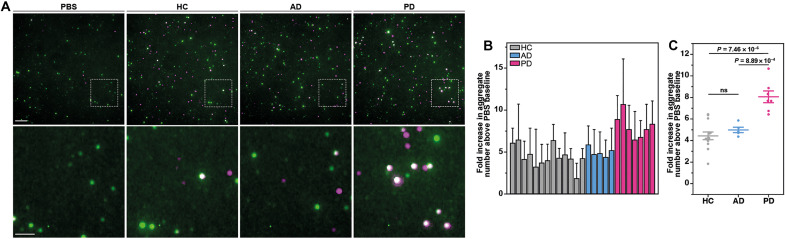
STAPull detects α-syn aggregates in PD patient CSF. (**A**) Representative STAPull images of in life CSF from patients diagnosed with PD, AD, or healthy control individuals (HC) and in comparison to a PBS-only control (as indicated). The boxed region indicated is enlarged in the row below. Monomeric species are visible as single-color puncta (green or magenta) and oligomeric as two-color (white). Scale bars, 5 μm (top) and 2 μm (bottom). (**B**) Quantification by case of aggregate density given as fold increase above PBS baseline. Error bars indicate SD of 64 technical repeats. (**C**) Dot plot comparing the data in (B) by diagnosis, where mean is indicated by long horizontal line and SE by vertical bars. Statistical significance between diagnostic groups was confirmed by one-way ANOVA (*P* value 9.88 × 10^−6^), where PD samples alone differed from other groups based on Tukey means comparison (*P* values indicated). ns, not significant.

Given the sensitivity of STAPull for the detection of secreted α-syn aggregates, we next sought to assess the approach for the detection of aggregates in patient antemortem CSF, which had been previously characterized by ELISA (*23*) and SAVE imaging (*6*) (demographics, clinical features, and biomarkers outlined in table S3). As STAPull is effective in minimizing background, 50 μl of CSF could be applied without dilution to maximize α-syn enrichment on the surface. CSF from patients with PD (*n* = 7), patients with AD (*n* = 5) or healthy individuals (*n* = 13) were exposed to a surface coated with SYN-CT1 antibody, which targets the early C-terminal region of α-syn, and subsequently probed with SYN-NT1, which targets the N-terminal region of α-syn. This pairing aimed to ensure detectability of extreme C-terminally truncated protein commonly associated with PD pathology (*24*). The data showed that, akin to published SAVE data (*6*), a significant increase in the α-syn aggregate number is observed for individuals diagnosed with PD [mean ± SEM: 8.05 ± 0.54 fold above phosphate-buffered saline (PBS) baseline aggregate number], as compared to healthy controls (4.44 ± 0.36 fold) giving an analysis of variance (ANOVA) *P* value of 7.47 × 10^−6^ (Fig. 6). It should be noted that some overlap was observed between the healthy control samples with the highest aggregate burden and the PD patient samples with the lowest aggregate burden. Further exploration will elucidate whether different antibody combinations may further delineate case from control or whether STAPull is detecting presymptomatic elevations in α-syn aggregates in certain control cases. STAPull did not detect a significant elevation in α-syn aggregates in the AD patient CSF (mean ± SEM: 4.98 ± 0.25, *P* value of 0.68) versus healthy control, and thus, unlike SAVE imaging, is able to specifically differentiate the elevated α-syn burden in PD patient biofluids (Fig. 6). Given this specificity, STAPull offers an exciting approach toward diagnosing underlying proteinopathy, which we anticipate will complement established biomarkers of generalized neurodegeneration (*25*).

## DISCUSSION

At present, neurodegenerative diseases are clinically diagnosed by the manifestation of symptoms that follow irreversible neuronal cell death. However, the neurodegenerative process begins 10 to 20 years before symptom onset, and such late diagnosis precludes the introduction of disease-modifying therapeutics when they could have the greatest effect. This issue is considered a major contributor to the failure of late-stage clinical trials (*26*) and underlines the need for biomarker-based diagnostic tools to detect neurodegeneration at its earliest stages.

Ensemble immunoassays such as ELISA or single molecule array are the gold-standard approach for protein biomarker detection, and their adaptation for aggregated protein pulldown have demonstrated oligomer-specific detection (*4*, *16*, *17*). However, these approaches detect oligomeric α-syn in the CSF of both healthy individuals (accounting for ~4% of total α-syn) and patients with PD (~9% of total α-syn), highlighting the need to dissect pathological from benign species, particularly in early-stage patients where elevation alone may be incremental. Techniques such as SAVE and SiMPull have attempted to address the need for aggregate species stratification through single-molecule visualization. SAVE imaging has observed different aggregate subpopulations in healthy versus disease cases based on SAVE intensity (*6*) but cannot specify protein identity. SiMPull is able to build on this by offering protein specificity alongside single-molecule detection; however, it lacks the sensitivity for diagnostic applications because of high background from monomeric protein and nonspecific binding.

We describe here an alternative approach, STAPull, to address these issues. STAPull enables single-molecule detection and differentiation of protein-specific oligomers, the earliest-stage aggregate species associated with neurotoxicity. We use detection of α-syn, the hallmark aggregating protein of synucleinopathies such as PD, to demonstrate picomolar sensitivity capable of detecting significantly higher oligomer burdens in PD patient CSF as compared to healthy control cases. This approach is broadly applicable to the detection of any proteinopathy or multimeric structures by replacement of the capture and detection antibodies. Furthermore, STAPull does not require the use of oligomer-specific antibodies that may only detect specific conformations but instead can be used with antibodies targeting the constituent protein.

The key advantage of STAPull is its ability to identify and visualize protein aggregates, providing opportunity for the discrimination of aggregate subspecies. As different oligomer strains have been identified in a number of neurodegenerative diseases, including PD, AD, and Creutzfeldt-Jakob disease (*27*–*29*), the detection of subpopulations with different pathological properties may provide a powerful prognostic biomarker. Toward this end, incorporation of super-resolution imaging techniques, such as DNA points accumulation for imaging in nanoscale topography or direct stochastic optical reconstruction microscopy, could enable oligomer stratification based on morphological metrics.

In summary, we present a technique that enables, to our knowledge, the first direct detection of specific protein aggregates at the single-molecule level. This tool has immediate implications for aiding the evaluation of aggregation-inhibiting drugs and clinical trial outcomes and may present opportunities for early-stage diagnostics.

## MATERIALS AND METHODS

### Preparation of α-syn

Wild-type α-syn [Addgene, 36046; (*30*)] was expressed and harvested from *Escherichia coli* as described previously (*31*). The cell pellet was resuspended in 20 mM tris-HCl (pH 8.0), 1 mM EDTA (buffer A) supplemented with 1 mM phenylmethylsulfonyl fluoride, lysed by 15-min pulsed probe sonication, and clarified by 12,000-RCF centrifugation at 4°C. The supernatant was recovered, heated to 80°C for 10 min, and centrifuged at 12,000 RCF. Streptomycin sulfate (10 mg/ml; 086 K1263) was added to the supernatant and incubated 20 min at 10°C with 100-rpm shaking. Following centrifugation at 12,000 RCF, the supernatant was collected and the previous step repeated with ammonium sulfate (360 mg/ml; 101575102). The pellet was collected by 45-min centrifugation at 12,000 RCF, resuspended in 25 ml of buffer A, and dialyzed overnight at 4°C in buffer A. The sample was loaded onto a HiTrap Q FF column (GE Healthcare Life Sciences) on an ÄKTA Start Protein Purification System (GE Healthcare Life Sciences). α-Syn was eluted at approximately 300 mM NaCl with a salt gradient from 0 to 1000 mM. The eluent was then loaded into a HiPrep 26/60 Sephacryl S-200 size exclusion column and eluted in 20 mM tris-HCl (pH 7.4) and 100 nM NaCl. Fractions corresponding to the ultraviolet chromatograph peak were recovered and confirmed by SDS–polyacrylamide gel electrophoresis (SDS-PAGE), then concentrated using a 5-kDa spin column (Sartorius, VSO611) and flash frozen with liquid nitrogen in single-use aliquots for storage at −80°C. The protein purity was quality checked by liquid chromatography–tandem mass spectrometry on a quadrupole ion-mobility time-of-flight instrument (Synapt G2, Waters Corp.).

### In vitro aggregation of α-syn

Monomeric wild-type α-syn was ultracentrifuged at 90,000 RCF for 1 hour at 4°C to remove any amorphous aggregates (Beckman Coulter Optima Max-XP with MLA-130 rotor). The supernatant was recovered, and the concentration was determined by absorbance at 275 nm using an extinction coefficient of 5600 M^−1^ cm^−1^. The concentration was adjusted to 70 μM by dilution in aggregation buffer (0.02-μm–filtered 20 mM tris-HCl (pH 7.4) and 100 nM NaCl, 0.01% (w/v) NaN_3_). The protein was then incubated at 37°C for 96 to 120 hours with constant agitation at 200 rpm, and samples were removed at regular intervals. The end-product was sonicated 10 min at 10°C (0.5 min on/0.5 min off) using a Biorupter Pico (Diagenode) and used in experiments unless otherwise stated. All aggregates were flash frozen with liquid nitrogen in single-use aliquots for storage at −80°C.

### Generation of α-syn kinetically stable oligomers and preformed fibrils

Human α-syn monomer, oligomers, and preformed fibrils are available from a commercial source (StressMarq Biosciences catalog no. SPR-321, SPR-484, and SPR-322, respectively), which are generated using methods previously described. Before oligomerization/fibrilization, α-syn monomer was thawed, mixed, and centrifuged 20,000*g* at 4°C to remove any pre-existing aggregate. The supernatant containing monomer was pooled and refilter sterilized (0.2 μm). To generate kinetically stable oligomers, monomeric α-syn was diluted to 2 mg/ml in 1× phosphate buffer (pH 7.4), evaporated in a Speedvac (Savant), and resuspended in dH_2_O a total of 50 cycles. Oligomers were pooled, washed on a 100-kDa molecular weight cutoff concentrator (Amicon), concentrated to 2 mg/ml, filter sterilized (0.2 μm), aliquoted, and frozen to −80°C. Oligomers were confirmed by native PAGE, size exclusion chromatography, and transmission electron microscopy (TEM). To generate preformed fibrils (PFFs), monomers were diluted to 5 mg/ml with PBS (pH 7.4) and shaken at 1000 rpm, 37°C, for 7 days using a Thermomixer C with a heated lid. PFFs were aliquoted, frozen at −80°C, and confirmed by sedimentation assay, ThT response, and TEM.

### Generation and aggregation of labeled α-synuclein

The Cys variant of wild-type α-syn was labeled as reported previously (*32*) with Alexa Fluor 488 C_5_ maleimide (AF488) and Alexa Fluor 647 C_2_ maleimide (AF647) (Thermo Fisher Scientific). A 70 μM solution made up of single-color or equimolar concentrations of AF488- and AF647-labeled α-syn prepared in 0.02-μm syringe-filtered (Anotop, Whatman) PBS (pH 7.4). The aggregation mixture was supplemented with 0.01% NaN_3_ to prevent bacterial growth and was incubated at 37°C with orbital shaking at 200 rpm for 48 hours. The product was either used directly for single-molecule FRET (smFRET) or flash frozen with LN_2_ in single-use aliquots for storage at −80°C.

### Aggregation of Aβ

Commercial Aβ 1—42 monomer (Anaspec, AS-20276) was prepared at 2 μM in saline sodium phosphate EDTA (SSPE) buffer and aggregated by 48-hour incubation at 37°C. Single-use aliquots were flash frozen in liquid nitrogen and stored at −80°C.

### Aggregation of tau

Tau4R monomer was diluted to 20 μM in SSPE buffer supplemented with 2 μM heparin and 0.01% (w/v) NaN_3_. Protein aggregation was carried out by 14-day incubation at 37°C, following which aliquots were flash frozen in liquid nitrogen and stored at −80°C.

### ThT preparation

ThT stock solutions were prepared by diluting ThT (Abcam, ab120751) into neat ethanol to give a final concentration ~5 M. The solution was vortexed thoroughly, and a working stock prepared at ~200 μM by dilution in PBS. The solution was filtered through a 0.02-μm syringe filter (Whatman, FIL2824) to remove insoluble ThT that may give rise to fluorescent puncta, and the concentration was confirmed by absorbance at 412 nm, using an extinction coefficient of 36,000 M^−1^ cm^−1^. The working stock was stored in the dark at 4°C and used for a maximum of 4 days with fresh filtration on day of use.

### Antibody labeling

All antibodies were generated by UCB Biopharma using single B cell technology and selected on the basis of affinity to human fibrillar α-syn as determined by surface plasmon resonance. Details of all antibodies are outlined in table S1. Stable antibody conjugation was carried out by reaction of the antibody primary amino groups with either NHS ester–linked AF488/AF647 dyes or NHS-PEG_4_-biotin for the detection and capture antibodies, respectively. For this purpose, commercial labeling kits were used (A20181/6, Thermo Fisher Scientific and Pierce, 90407, respectively) in accordance with the manufacturer’s instructions. The concentration of the resultant conjugate was determined on the basis of absorbance at 280 nm, using an extinction coefficient of 210,000 M^−1^ cm^−1^, corrected in the case of the dye-labeled conjugates using absorbance at 494 or 650 nm as applicable as per the manufacturer’s instructions.

### STAPull surface preparation

Borosilicate glass coverslips (24 mm by 60 mm; VWR, 631-1339) were exposed to argon plasma for 45 min to remove organic contaminants. They were subsequently submerged in 0.22-μm–filtered 1 M KOH for 20 min to activate the surface for silane functionalization, rinsed in deionized water, and transferred to 1% (3-aminopropyl) trimethoxysilane (Sigma-Aldrich, 281778) in methanol supplemented with 5% acetic acid for 20-min incubation in the dark. Following silanization, the coverslips were serially washed in methanol and deionized water, blast dried with argon gas, and affixed to an 18-well gasket (Ibidi, 81818). Freshly prepared PEG solution (50 μl) [mPEG-Succinimidyl Varelate (SVA) (100 mg/ml; 5000 Da, Laysan Bio), biotin-mPEG-SVA (5 mg/ml; 5000 Da, Laysan Bio), and 0.1 M NaHCO_3_] was applied to each well and incubated overnight. The surface was then rinsed with deionized water and blast dried with argon gas, before incubating 10 min with streptavidin (0.2 mg/ml; Thermo Fisher Scientific, 21125) in 0.02-μm–filtered T50 buffer [10 mM tris-HCl (pH 8.0) supplemented with 50 mM NaCl] and subsequently washed three times in T50.

To specifically enrich the target protein on the surface, 100 nM of appropriate capture antibody diluted in 0.02-μm–filtered PBS was applied for 20-min incubation (SYN-CT1-biot for α-syn and anti–tau-biot for tau pulldown). Excess antibody was removed by washing three times with PBS and 50-μl sample applied. Unless otherwise stated, recombinant protein generated in-house was applied at 10 nM concentration for 20 min, commercially sourced α-syn monomer, oligomer, and fibrils (Stressmarq, SPR-321, SPR-484, and SPR-322) were applied at 25 nM concentration supplemented with 1% bovine serum albumin, conditioned media was applied neat for 20 min, and randomized CSF applied neat for 24 hours at room temperature (RT). Wells were washed three times with PBS to remove unbound protein and incubated with a 1:1 mixture of 1 nM AF488- and 1 nM AF647-labeled detection antibody diluted in T50 for tris-mediated quenching of any remaining unbound dye. Detection antibodies used were anti-tau (10 min at RT) to probe tau protein, SYN-NT1 (24 hours at RT) to probe CSF samples, and SYN-CT2 (10 min at RT) in all other cases. The lower-affinity SYN-NT1 antibody required a longer incubation but allows detection of any captured extreme C-terminally truncated α-syn protein. Excess detection antibody was removed by washing three times with PBS.

### Surface preparation for SAVE imaging

Coverslip surfaces were prepared as described previously (*6*). Briefly, coverslips were exposed to argon plasma for 45 min to remove autofluorescent organic contaminants. Frame-seal slide chambers (Biorad, SLF-0601) were affixed to the glass, and 50 μl of PLL (Sigma-Aldrich, P4707-50ML) was incubated in the chamber for 15 min. Excess PLL was removed by washing three times with 0.02-μm–filtered PBS. Protein (500 nM) was incubated on the surface for 1 hour and replaced with 5 μM ThT solution immediately before imaging.

### Single-molecule imaging

All samples were imaged using an ONI Nanoimager equipped with a 100×/1.4 numerical aperture oil immersion objective lens and ORCA-Flash 4.0 V3 scientific complementary metal-oxide semiconductor camera. Samples were exposed sequentially to 638-, 488-, and/or 405-nm excitation by total internal reflection using a 53.5° illumination angle for visualization of AF647, AF488, and ThT as required. The resultant emission was split with a 640-nm dichroic, projecting emission above and below this wavelength onto different regions of the camera chip for dual-channel viewing. Each field of view (FOV) was imaged for 10 frames at a rate of 20 frames s^−1^, and an 8 × 8 grid of 200-μm–spaced FOVs was captured per condition to account for any region-specific variations.

### Image preprocessing

Single-channel images were cropped from the dual-view acquisition and registered on the basis of ground-truth TetraSpeck microsphere (Thermo Fisher Scientific, T7279) data captured alongside the dataset. The imreg_dft Python library (*33*) was used to perform and automate channel registration. Single-channel image series were subsequently maximum intensity projected to reduce image noise.

### STAPull two-color coincidence analysis

Particle detection, colocalization, and quantification were performed using the ComDet v.0.5.5 plugin for ImageJ (https://github.com/ekatrukha/ComDet). To exclude background fluorescence variations, detections smaller than the resolution limit of the technique were excluded and an intensity threshold set to 5 SD above image mean was used. This value was determined empirically by assessing the number of events detected in the presence and absence of detection antibody (fig. S8). Two-color events with a center of mass within two-pixel proximity were considered coincident. To assess chance coincidence due to sample density, the same measurements were applied to a transformed control of each FOV, whereby one channel was flipped horizontally and vertically with respect to the other. Presented aggregate values represent the mean coincidence of all FOVs less their chance detections, calibrated to the imaged area. Where reported, total protein values represent the sum of single channel events, less the coincident.

### SiMPull threshold analysis

Single-channel estimations of aggregated species were carried out using the AF488 channel. A threshold intensity value was subtracted from all pixels of the image to exclude signal that is unlikely to originate from oligomeric species. This value was calculated as 3 SDs above the mean pixel signal of all noncoincident species identified via STAPull analysis. Particle detection and quantification were subsequently carried out using the ComDet v.0.5.5 plugin for ImageJ.

### LoD analysis

α-Syn aggregate concentration series (100 fM to 10 nM) were prepared in triplicate in 0.02-μm–filtered PBS (Thermo Fisher Scientific, 10209252) and the resultant aggregate numbers quantified by STAPull and SiMPull. For comparison to ThT fluorimetry, an additional 0.1 to 7 μM concentration series was prepared in 5 μM ThT-supplemented PBS and their fluorescence intensity measured using a Denovix DS-11 Fxt Fluorimeter with 470-nm excitation and 514- to 567-nm emission detection. Concentration calibration curves were generated using the mean detections and fit with a second-order polynomial using OriginPro software (OriginLab). The limit of blank (LoB), which indicates the highest number of counts expected when no analyte is detected, was calculated as (*14*)

$$\mathrm{LoB}={\mathrm{mean}}_{\mathrm{blank}}+1.645({\mathrm{SD}}_{\mathrm{blank}})$$

where the blank sample contained no protein. The LoD, which denotes the lowest analyte concentration likely to be reliably distinguished from the LoB, was subsequently calculated as

$$\mathrm{LoD}=\mathrm{LoB}+1.645({\mathrm{SD}}_{\mathrm{lowest}\;\mathrm{concentration}})$$

and the equivalent concentration derived from the standard curve equation. We report aggregate concentration based on the equivalent monomer starting concentration, which overestimates the oligomer fraction. The oligomer concentration was thus estimated as

$${\mathrm{Conc}}_{\mathrm{olig}}={\mathrm{Conc}}_{\mathrm{mon}}\times \left(\frac{\text{Fraction oligomeric}}{\text{Mean monomer incorporation}}\right)$$

where fraction oligomeric is the proportion of total STAPull detections that were coincident, and mean monomer incorporation is the average coincident signal normalized by average noncoincident signal, both determined with a 5 nM α-syn aggregate sample.

### SAVE analysis

SAVE image analysis was performed using custom-written python code. Single-channel image series were mean intensity projected, and a rolling background subtraction was applied. Local background was computed over an 11 × 11 kernel as the mean value less the global baseline as given by the intensity of the lowest 1% of pixels. The image was thresholded and binarized, using a threshold value equal to 3 SDs above the mean image signal when no protein or monomeric protein alone is present, and resulting features counted.

### Differentiation and culture of iPSC-derived mDA neurons

The *SNCA* tripication iPS cell line, AST18, was derived from skin fibroblasts donated by a patient with PD harboring a triplication of a region on *Chr4q22* encompassing the *SNCA* locus (*34*). A full knockout of all four *SNCA* copies, AST18-7B, was derived from the parent triplication line via CRISPR (*35*). iPS cell culture and directed differentation protocols used to generate mDA neuons were described previously (*35*). Successful differentiation of mDA neurons was confirmed by probing cell type-specific identity markers (fig. S9).

### CSF samples

The CSF samples used in this study were obtained from UCL Queen Square Institute of Neurology. Informed consent was obtained from all participants, including access to their clinical data. No additional ethics approval was required for our use of the CSF after obtaining it from the UCL Queen Square Institute of Neurology. CSF was collected by lumbar puncture from 7 patients with clinically defined PD (aged 53 to 75, mean ± SD = 64 ± 7), 5 patients with clinically defined AD (aged 43 to 68, mean ± SD = 60 ± 10), and 13 healthy individuals (aged 46 to 76, mean ± SD = 63 ± 7) based on internationally established criteria (*36*, *37*). Samples were initially collected by UCL Queen Square Institute of Neurology for a previous biomarker study (*23*). Full demographic, clinical and biomarker information is outlined in table S2. Sample collection and storage followed a standardized procedure (www.neurochem.gu.se/TheAlzAssQCProgram). Briefly, lumbar puncture was performed between 09:00 and 12:00. CSF (15 ml) was collected in sterile polypropylene tubes. The collected CSF samples were gently mixed to avoid gradient effects and centrifuged at 4000 rpm for 10 min at 4°C before dividing into 0.5-ml aliquots in Protein LoBind tubes, which were frozen on dry ice and stored at −80°C. Blood-contaminated samples (>500 red blood cells per microliter) were excluded. Time between sample collection, centrifugation, and freezing was maximum 1 hour. The study was conducted in accordance with local clinical research regulations and with the provisions of the Helsinki declaration. Informed consent was obtained from all participants at the time of collection, including access to their clinical data, and no additional ethics approval was required for our use of the material.

### Single-molecule FRET

Forty-eight–hour aggregates of equimolar AF488- and AF647-labeled α-syn was first diluted by a factor of 1:500 into PBS (pH 7.4), and subsequently by a factor of 1:500 into PBS (pH 7.5) containing either no antibody or 2 nM of SYN-NT1, SYN-CT1, or SYN-CT2 antibody. The solution was loaded into a 200-μl gel-loading tip (Thermo Fisher Scientific) attached to the inlet port of a microfluidic channel (25 μm in height, 100 μm in width, and 1 cm in length) (*38*) mounted onto a single-molecule confocal microscope. The confocal volume was focused 10 μm into the center of the channel, and the solution was passed through the channel at an average velocity of 1 cm/s by applying a negative pressure generated using a syringe pump (KD Scientific, 78,8101) attached to the outlet port via polyethylene tubing [^1^/_32_-in (0.79-mm) inner diameter, ^1^/_16_-in (1.59-mm) outer diameter; Darwin Microfluidics, Hythe, Paris, France].

Confocal microscopy measurements were performed on a custom built single-molecule instrument described previously (*11*, *39*). The sample was illuminated with 488-nm radiation (6.35 mW at the back port of the microscope), and the FRET donor (AF488) and acceptor (AF647) photons were detected as oligomers transited the confocal volume over a measurement time of 900 s. The photons from each channel were combined into time bins of 100 μs, the expected residence time of the molecules in the confocal volume.

Analysis of the data was performed as described previously (*39*) using custom written python code (DOI: 10.5281/zenodo.8189826). Coincident events, corresponding to oligomers containing both fluorophores, were those in which at least 8 photon counts bin^−1^ were detected in each channel. After accounting for autofluorescence and cross-talk (Eqs. 1 and 2), the FRET efficiency and approximate size of each oligomer were calculated from the intensities in each channel (Eqs. 3 and 4)

$$I_{D}=D-A_{D}$$ (1)

$$I_{A}=A-A_{A}-C\times D$$ (2)

$$E=\frac{I_{A}}{I_{A}+I_{D}}$$ (3)

$$\text{Approximate size}=\frac{2(I_{D}+I_{A})}{I_{\mathrm{monomer}}}$$ (4)

where *D* and *A* are the intensities from the donor and acceptor channels with donor excitation only, respectively; *A*_D_ and *A*_A_ are the autofluorescence in the donor and acceptor channels, measured in the absence of fluorophores; *C* is the instrument-specific cross-talk from the donor to acceptor channel; and *I*_monomer_ is the average monomer brightness, calculated from the average of the noncoincident donor channel bursts intensities.

Oligomers were divided into three groups depending on their approximate size. The smallest oligomers correspond to <5-mers, and the remaining are classified as medium in size (up to 150 monomer units per oligomer). The division into type A and type B oligomers was made by globally fitting the medium-sized oligomer FRET efficiency histograms (shared x-center and x-width for all time points and treatments) to two Gaussian distributions (type A has a lower FRET efficiency than type B), which were then integrated to determine the event rate for each population. The global fitting ensures that the same populations are identified from each dataset. Two-dimensional contour plots of size and FRET efficiency were generated in Igor Pro 7 (Wavemetrics).

### Statistical analysis

Statistical analysis was carried out using GraphPad Prism, R, or OriginPro. A Shapiro-Wilk test and *F* test were used to confirm normal distribution and equal variance, respectively. If these assumptions were satisfied, statistical significance was ascertained for grouped data using a one-way ANOVA with Tukey post hoc means comparison or for pairwise comparison using an unpaired Student *t* test. Grouped data that did not satisfy these assumptions used a Kruskal-Wallis test with Dunn’s test post hoc means comparison to assess statistical significance.

## Supplementary Materials

### This PDF file includes:

Figs. S1 to S9 Tables S1 and S2 Legend for table S3 Legend for data S1 References

## References

[1] F. Chiti, C. M. Dobson, Protein misfolding, functional amyloid, and human disease. Annu. Rev. Biochem. 75, 333–366 (2006). 16756495 10.1146/annurev.biochem.75.101304.123901

[2] B. Winner, R. Jappelli, S. K. Maji, P. A. Desplats, L. Boyer, S. Aigner, C. Hetzer, T. Loher, M. Vilar, S. Campioni, C. Tzitzilonis, A. Soragni, S. Jessberger, H. Mira, A. Consiglio, E. Pham, E. Masliah, F. H. Gage, R. Riek, In vivo demonstration that α-synuclein oligomers are toxic. Proc. Natl. Acad. Sci. U.S.A. 108, 4194–4199 (2011). 21325059 10.1073/pnas.1100976108 PMC3053976

[3] C. A. Lasagna-Reeves, D. L. Castillo-Carranza, U. Sengupta, A. L. Clos, G. R. Jackson, R. Kayed, Tau oligomers impair memory and induce synaptic and mitochondrial dysfunction in wild-type mice. Mol. Neurodegener. 6, 39 (2011). 21645391 10.1186/1750-1326-6-39 PMC3224595

[4] T. Tokuda, M. M. Qureshi, M. T. Ardah, S. Varghese, S. A. S. Shehab, T. Kasai, N. Ishigami, A. Tamaoka, M. Nakagawa, O. M. A. El-Agnaf, Detection of elevated levels of α-synuclein oligomers in CSF from patients with Parkinson disease. Neurology 75, 1766–1772 (2010). 20962290 10.1212/WNL.0b013e3181fd613b

[5] L. C. Walker, Proteopathic strains and the heterogeneity of neurodegenerative diseases. Annu. Rev. Genet. 50, 329–346 (2016). 27893962 10.1146/annurev-genet-120215-034943 PMC6690197

[6] M. H. Horrocks, S. F. Lee, S. Gandhi, N. K. Magdalinou, S. W. Chen, M. J. Devine, L. Tosatto, M. Kjaergaard, J. S. Beckwith, H. Zetterberg, M. Iljina, N. Cremades, C. M. Dobson, N. W. Wood, D. Klenerman, Single-molecule imaging of individual amyloid protein aggregates in human biofluids. ACS Chem. Nerosci. 7, 399–406 (2016). 10.1021/acschemneuro.5b00324 PMC4800427 26800462

[7] M. J. Morten, L. Sirvio, H. Rupawala, E. Mee Hayes, A. Franco, C. Radulescu, L. Ying, S. J. Barnes, A. Muga, Y. Ye, Quantitative super-resolution imaging of pathological aggregates reveals distinct toxicity profiles in different synucleinopathies. Proc. Natl. Acad. Sci. U.S.A. 119, e2205591119 (2022). 36206368 10.1073/pnas.2205591119 PMC9573094

[8] J.-E. Lee, J. C. Sang, M. Rodrigues, A. R. Carr, M. H. Horrocks, S. De, M. N. Bongiovanni, P. Flagmeier, C. M. Dobson, D. J. Wales, S. F. Lee, D. Klenerman, Mapping surface hydrophobicity of α-synuclein oligomers at the nanoscale. Nano Lett. 18, 7494–7501 (2018). 30380895 10.1021/acs.nanolett.8b02916 PMC6295917

[9] A. Jain, R. Liu, B. Ramani, E. Arauz, Y. Ishitsuka, K. Ragunathan, J. Park, J. Chen, Y. K. Xiang, T. Ha, Probing cellular protein complexes using single-molecule pull-down. Nature 473, 484–488 (2011). 21614075 10.1038/nature10016 PMC3103084

[10] G. Je, B. Croop, S. Basu, J. Tang, K. Y. Han, Y.-S. Kim, Endogenous α-synuclein protein analysis from human brain tissues using single-molecule pull-down assay. Anal. Chem. 89, 13044–13048 (2017). 29172450 10.1021/acs.analchem.7b04335

[11] A. Chappard, C. Leighton, R. S. Saleeb, K. Jeacock, S. R. Ball, K. Morris, O. Kantelberg, J.-E. Lee, E. Zacco, A. Pastore, M. Sunde, D. J. Clarke, P. Downey, T. Kunath, M. H. Horrocks, Single-molecule two-color coincidence detection of unlabeled α-synuclein aggregates. Angew. Chem. Int. Ed. Engl. 62, e202216771 (2023). 36762870 10.1002/anie.202216771 PMC10946743

[12] L. Tosatto, M. H. Horrocks, A. J. Dear, T. P. J. Knowles, M. Dalla Serra, N. Cremades, C. M. Dobson, D. Klenerman, Single-molecule FRET studies on α-synuclein oligomerization of Parkinson’s disease genetically related mutants. Sci. Rep. 5, 16696 (2015). 26582456 10.1038/srep16696 PMC4652217

[13] A. A. Hariri, S. S. Newman, S. Tan, D. Mamerow, A. M. Adams, N. Maganzini, B. L. Zhong, M. Eisenstein, A. R. Dunn, H. T. Soh, Improved immunoassay sensitivity and specificity using single-molecule colocalization. Nat. Commun. 13, 5359 (2022). 36097164 10.1038/s41467-022-32796-x PMC9468026

[14] D. A. Armbruster, T. Pry, Limit of blank, limit of detection and limit of quantitation. Clin. Biochem. Rev. 29, S49–S52 (2008). 18852857 PMC2556583

[15] S. W. Chen, S. Drakulic, E. Deas, M. Ouberai, F. A. Aprile, R. Arranz, S. Ness, C. Roodveldt, T. Guilliams, E. J. De-Genst, D. Klenerman, N. W. Wood, T. P. J. Knowles, C. Alfonso, G. Rivas, A. Y. Abramov, J. M. Valpuesta, C. M. Dobson, N. Cremades, Structural characterization of toxic oligomers that are kinetically trapped during α-synuclein fibril formation. Proc. Natl. Acad. Sci. 112, E1994–E2003 (2015). 25855634 10.1073/pnas.1421204112 PMC4413268

[16] I. van Steenoven, N. K. Majbour, N. N. Vaikath, H. W. Berendse, W. M. van der Flier, W. D. J. van de Berg, C. E. Teunissen, A. W. Lemstra, O. M. A. El-Agnaf, α-Synuclein species as potential cerebrospinal fluid biomarkers for dementia with lewy bodies. Mov. Disord. 33, 1724–1733 (2018). 30440090 10.1002/mds.111 PMC6519232

[17] N. K. Majbour, N. N. Vaikath, K. D. van Dijk, M. T. Ardah, S. Varghese, L. B. Vesterager, L. P. Montezinho, S. Poole, B. Safieh-Garabedian, T. Tokuda, C. E. Teunissen, H. W. Berendse, W. D. J. van de Berg, O. M. A. El-Agnaf, Oligomeric and phosphorylated α-synuclein as potential CSF biomarkers for Parkinson’s disease. Mol. Neurodegener. 11, 7 (2016). 26782965 10.1186/s13024-016-0072-9 PMC4717559

[18] M. Biancalana, S. Koide, Molecular mechanism of Thioflavin-T binding to amyloid fibrils. Biochim. Biophys. Acta 1804, 1405–1412 (2010). 20399286 10.1016/j.bbapap.2010.04.001 PMC2880406

[19] N. Lorenzen, S. B. Nielsen, A. K. Buell, J. D. Kaspersen, P. Arosio, B. S. Vad, W. Paslawski, G. Christiansen, Z. Valnickova-Hansen, M. Andreasen, J. J. Enghild, J. S. Pedersen, C. M. Dobson, T. P. J. Knowles, D. E. Otzen, The role of stable α-synuclein oligomers in the molecular events underlying amyloid formation. J. Am. Chem. Soc. 136, 3859–3868 (2014). 24527756 10.1021/ja411577t

[20] A. B. Singleton, M. Farrer, J. Johnson, A. Singleton, S. Hague, J. Kachergus, M. Hulihan, T. Peuralinna, A. Dutra, R. Nussbaum, S. Lincoln, A. Crawley, M. Hanson, D. Maraganore, C. Adler, M. R. Cookson, M. Muenter, M. Baptista, D. Miller, J. Blancato, J. Hardy, K. Gwinn-Hardy, α-Synuclein locus triplication causes Parkinson's disease. Science 302, 841 (2003). 14593171 10.1126/science.1090278

[21] M. Iljina, G. A. Garcia, M. H. Horrocks, L. Tosatto, M. L. Choi, K. A. Ganzinger, A. Y. Abramov, S. Gandhi, N. W. Wood, N. Cremades, C. M. Dobson, T. P. J. Knowles, D. Klenerman, Kinetic model of the aggregation of α-synuclein provides insights into prion-like spreading. Proc. Natl. Acad. Sci. U.S.A. 113, E1206–E1215 (2016). 26884195 10.1073/pnas.1524128113 PMC4780632

[22] D. R. Whiten, Y. Zuo, L. Calo, M. L. Choi, S. De, P. Flagmeier, D. C. Wirthensohn, F. Kundel, R. T. Ranasinghe, S. E. Sanchez, D. Athauda, S. F. Lee, C. M. Dobson, S. Gandhi, M. G. Spillantini, D. Klenerman, M. H. Horrocks, Nanoscopic characterisation of individual endogenous protein aggregates in human neuronal cells. Chembiochem 19, 2033–2038 (2018). 30051958 10.1002/cbic.201800209 PMC6220870

[23] N. K. Magdalinou, R. W. Paterson, J. M. Schott, N. C. Fox, C. Mummery, K. Blennow, K. Bhatia, H. R. Morris, P. Giunti, T. T. Warner, R. de Silva, A. J. Lees, H. Zetterberg, A panel of nine cerebrospinal fluid biomarkers may identify patients with atypical parkinsonian syndromes. J. Neurol. Neurosurg. Psychiatry 86, 1240–1247 (2015). 25589779 10.1136/jnnp-2014-309562 PMC4564944

[24] Z. A. Sorrentino, B. I. Giasson, The emerging role of α-synuclein truncation in aggregation and disease. J. Biol. Chem. 295, 10224–10244 (2020). 32424039 10.1074/jbc.REV120.011743 PMC7383394

[25] L. Gaetani, K. Blennow, P. Calabresi, M. Di Filippo, L. Parnetti, H. Zetterberg, Neurofilament light chain as a biomarker in neurological disorders. J. Neurol. Neurosurg. Psychiatry 90, 870–881 (2019). 30967444 10.1136/jnnp-2018-320106

[26] D. Athauda, T. Foltynie, Challenges in detecting disease modification in Parkinson’s disease clinical trials. Parkinsonism Relat. Disord. 32, 1–11 (2016). 27499048 10.1016/j.parkreldis.2016.07.019

[27] L. Bousset, L. Pieri, G. Ruiz-Arlandis, J. Gath, P. H. Jensen, B. Habenstein, K. Madiona, V. Olieric, A. Böckmann, B. H. Meier, R. Melki, Structural and functional characterization of two α-synuclein strains. Nat. Commun. 4, 2575 (2013). 24108358 10.1038/ncomms3575 PMC3826637

[28] P. Liu, M. N. Reed, L. A. Kotilinek, M. K. O. Grant, C. L. Forster, W. Qiang, S. L. Shapiro, J. H. Reichl, A. C. A. Chiang, J. L. Jankowsky, C. M. Wilmot, J. P. Cleary, K. R. Zahs, K. H. Ashe, Quaternary structure defines a large class of amyloid-β oligomers neutralized by sequestration. Cell Rep. 11, 1760–1771 (2015). 26051935 10.1016/j.celrep.2015.05.021 PMC4494129

[29] Z. Krejciova, J. Alibhai, C. Zhao, R. Krencik, N. M. Rzechorzek, E. M. Ullian, J. Manson, J. W. Ironside, M. W. Head, S. Chandran, Human stem cell-derived astrocytes replicate human prions in a *PRNP* genotype-dependent manner. J. Exp. Med. 214, 3481–3495 (2017). 29141869 10.1084/jem.20161547 PMC5716027

[30] K. E. Paleologou, A. W. Schmid, C. C. Rospigliosi, H.-Y. Kim, G. R. Lamberto, R. A. Fredenburg, P. T. Lansbury Jr., C. O. Fernandez, D. Eliezer, M. Zweckstetter, H. A. Lashuel, Phosphorylation at Ser-129 but not the phosphomimics S129E/D inhibits the fibrillation of α-synuclein. J. Biol. Chem. 283, 16895–16905 (2008). 18343814 10.1074/jbc.M800747200 PMC2423264

[31] W. Hoyer, T. Antony, D. Cherny, G. Heim, T. M. Jovin, V. Subramaniam, Dependence of α-synuclein aggregate morphology on solution conditions. J. Mol. Biol. 322, 383–393 (2002). 12217698 10.1016/s0022-2836(02)00775-1

[32] N. Cremades, S. I. A. Cohen, E. Deas, A. Y. Abramov, A. Y. Chen, A. Orte, M. Sandal, R. W. Clarke, P. Dunne, F. A. Aprile, C. W. Bertoncini, N. W. Wood, T. P. J. Knowles, C. M. Dobson, D. Klenerman, Direct observation of the interconversion of normal and toxic forms of α-synuclein. Cell 149, 1048–1059 (2012). 22632969 10.1016/j.cell.2012.03.037 PMC3383996

[33] B. S. Reddy, B. N. Chatterji, An FFT-based technique for translation, rotation, and scale-invariant image registration. IEEE Trans. Image Process. 5, 1266–1271 (1996). 18285214 10.1109/83.506761

[34] M. J. Devine, M. Ryten, P. Vodicka, A. J. Thomson, T. Burdon, H. Houlden, F. Cavaleri, M. Nagano, N. J. Drummond, J.-W. Taanman, A. H. Schapira, K. Gwinn, J. Hardy, P. A. Lewis, T. Kunath, Parkinson’s disease induced pluripotent stem cells with triplication of the α-synuclein locus. Nat. Commun. 2, 440 (2011). 21863007 10.1038/ncomms1453 PMC3265381

[35] Y. Chen, K. S. Dolt, M. Kriek, T. Baker, P. Downey, N. J. Drummond, M. A. Canham, A. Natalwala, S. Rosser, T. Kunath, Engineering synucleinopathy-resistant human dopaminergic neurons by CRISPR-mediated deletion of the *SNCA* gene. Eur. J. Neurosci. 49, 510–524 (2019). 30472757 10.1111/ejn.14286 PMC6492083

[36] G. McKhann, D. Drachman, M. Folstein, R. Katzman, D. Price, E. M. Stadlan, Clinical diagnosis of Alzheimer’s disease: Report of the NINCDS-ADRDA Work Group under the auspices of Department of Health and Human Services Task Force on Alzheimer's disease. Neurology 34, 939–944 (1984). 6610841 10.1212/wnl.34.7.939

[37] National Collaborating Centre for Chronic Conditions (UK), *Parkinson’s Disease: National Clinical Guideline for Diagnosis and Management in Primary and Secondary Care* (Royal College of Physicians, 2006). 21089238

[38] M. H. Horrocks, L. Tosatto, A. J. Dear, G. A. Garcia, M. Iljina, N. Cremades, M. Dalla Serra, T. P. J. Knowles, C. M. Dobson, D. Klenerman, Fast flow microfluidics and single-molecule fluorescence for the rapid characterization of α-synuclein oligomers. Anal. Chem. 87, 8818–8826 (2015). 26258431 10.1021/acs.analchem.5b01811

[39] M. L. Choi, A. Chappard, B. P. Singh, C. Maclachlan, M. Rodrigues, E. I. Fedotova, A. V. Berezhnov, S. De, C. J. Peddie, D. Athauda, G. S. Virdi, W. Zhang, J. R. Evans, A. I. Wernick, Z. S. Zanjani, P. R. Angelova, N. Esteras, A. Y. Vinokurov, K. Morris, K. Jeacock, L. Tosatto, D. Little, P. Gissen, D. J. Clarke, T. Kunath, L. Collinson, D. Klenerman, A. Y. Abramov, M. H. Horrocks, S. Gandhi, Pathological structural conversion of α-synuclein at the mitochondria induces neuronal toxicity. Nat. Neurosci. 25, 1134–1148 (2022). 36042314 10.1038/s41593-022-01140-3 PMC9448679

[40] F. W. Doane, N. Anderson, *Diagnostic Virology—A Practical Guide And Atlas* (Cambridge Univ. Press, 1997).

[41] S. T. Kumar, S. Jagannath, C. Francois, H. Vanderstichele, E. Stoops, H. A. Lashuel, How specific are the conformation-specific α-synuclein antibodies? Characterization and validation of 16 α-synuclein conformation-specific antibodies using well-characterized preparations of α-synuclein monomers, fibrils and oligomers with distinct structures and morphology. Neurobiol. Dis. 146, 105086 (2020). 32971232 10.1016/j.nbd.2020.105086

