# Supplementary material for: Two-color coincidence single-molecule pulldown for the specific detection of disease-associated protein aggregates
Source: Sci Adv. 2023 Nov 15;9(46):eadi7359. doi: 10.1126/sciadv.adi7359 (PMC10651132; doi:10.1126/sciadv.adi7359)
Supplement: Supplementary file 1 — Figs. S1 to S9 Tables S1 and S2 Legend for table S3 Legend for data S1 References [file sciadv.adi7359_sm.pdf]

Science Advances
AAAS

Supplementary Materials for

Two-color coincidence single-molecule pulldown for the specific detection of disease-associated protein aggregates

Rebecca S. Saleeb *et al.*

Corresponding author: Mathew H. Horrocks, mathew.horrocks@ed.ac.uk

Sci. Adv. **9**, eadi7359 (2023)
DOI: 10.1126/sciadv.adi7359

The PDF file includes:

Figs. S1 to S9
Tables S1 and S2
Legend for table S3
Legend for data S1
References

Other Supplementary Material for this manuscript includes the following:

Table S3
Data S1

Fig. S1.

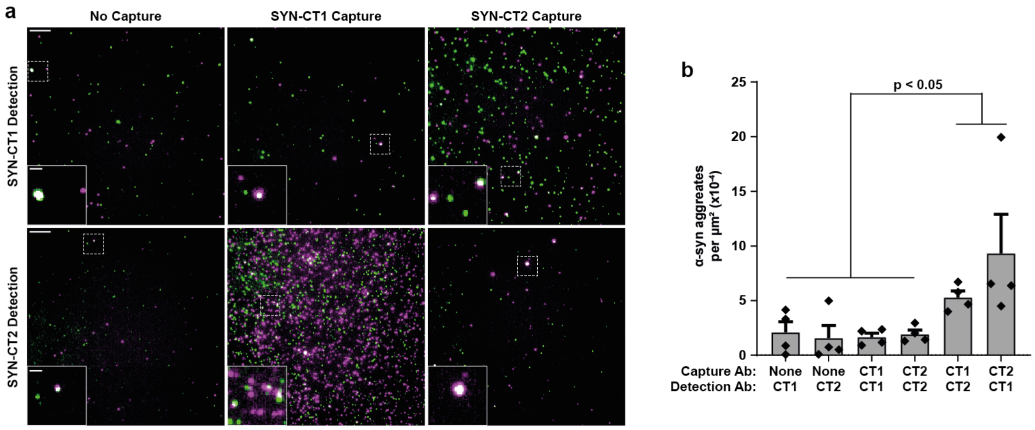

Fig. S1. STAPull sensitivity can be improved when different capture and detection antibodies are used. a Representative fluorescence images of 10 nM α-syn subjected to conditions favoring aggregation for 48h, immobilized and visualized using different combinations of the SYN-CT1 and SYN-CT2 antibodies for capture and detection. **b** Quantification of colocalized events (mean ± SEM, n=4, 16 FOV, total area = 64,119.71 μm^2). * $P < 0.05$ One-way ANOVA with Tukey multiple comparison test. (Note outlier in CT2 capture + CT1 detection sample was excluded for statistical analysis). Scale bars are 5 µm and 1 µm in length, for full view and inset images, respectively.

Fig. S2.

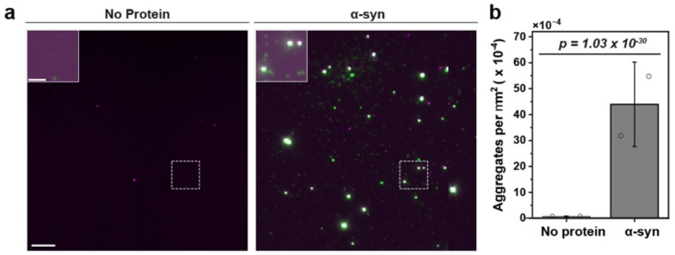

Fig. S2. α-syn STAPull using commercially-sourced antibodies. a Representative STAPull images in the presence (right) and absence (left) of 10 nM 96 hr α-syn aggregates using a surface coated with biotinylated 100 nM LB509 anti-α-syn and probed with a 1:1 ratio of Alexa Fluor 488 and Alexa Fluor 647 labeled 400 pM MJFR-14-6-4-2 anti-α-syn. Indicated region shown in inset at high-contrast and with a gamma of 0.5 applied to visualize any low-level signal. Scale bars are 5 µm in full frame, 2 µm in inset. **b** STAPull coincident events per µm^2 for the dataset presented in a (mean ± SD, n = 2, 64 technical repeats). Statistical analysis carried out with an unpaired student t-test demonstrates significantly higher aggregate numbers in the presence of α-syn.

Fig. S3.

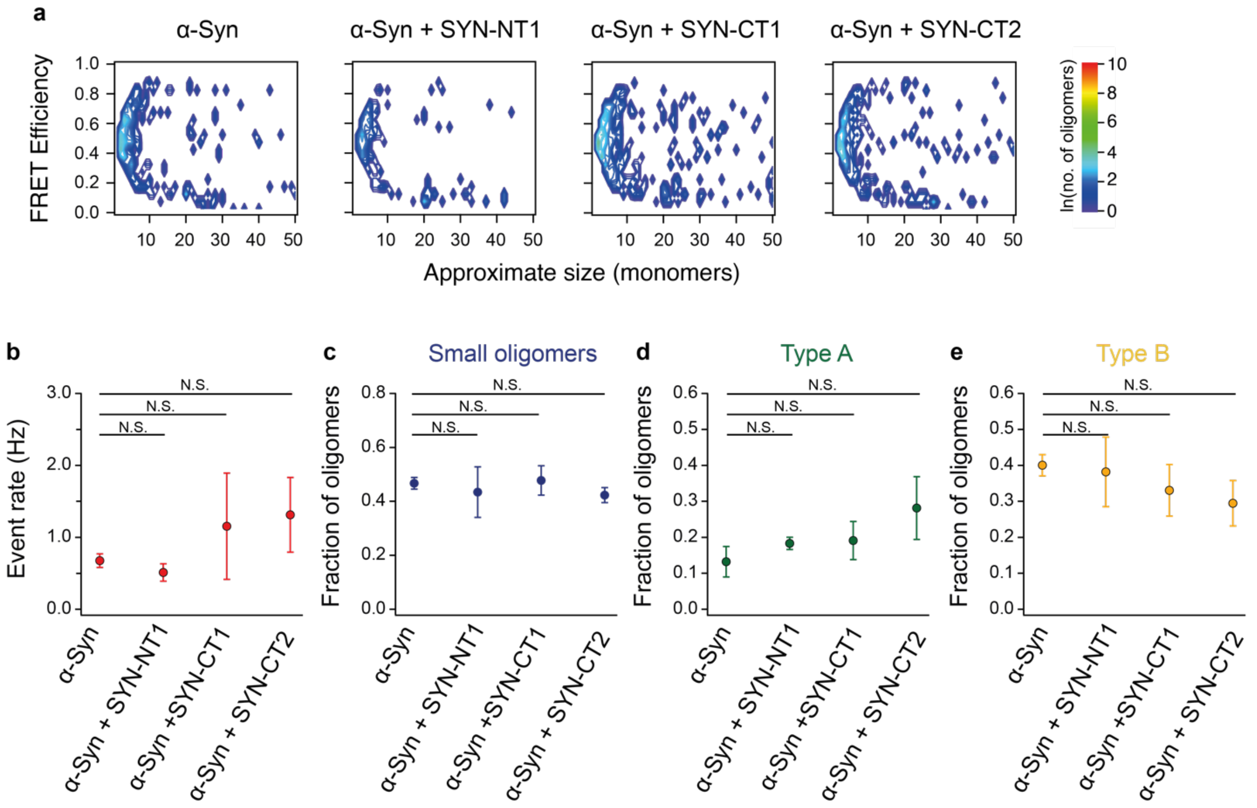

Fig. S3. Antibodies have no significant effect on the concentration or structure of α-syn oligomers. a Representative contour plots of size and FRET efficiency of labeled α-syn oligomers incubated without any antibody, or with 2 nM SYN-NT1, SYN-CT1, or SYN-CT2. **b** The rate of detected oligomers incubated with or without antibodies. The oligomer population was separated into small (**c**), Type A (**d**), or Type B (**e**) for each incubation. Error bars show standard deviation (n = 3) and statistical significance determined with an unpaired student t-test.

Fig. S4.

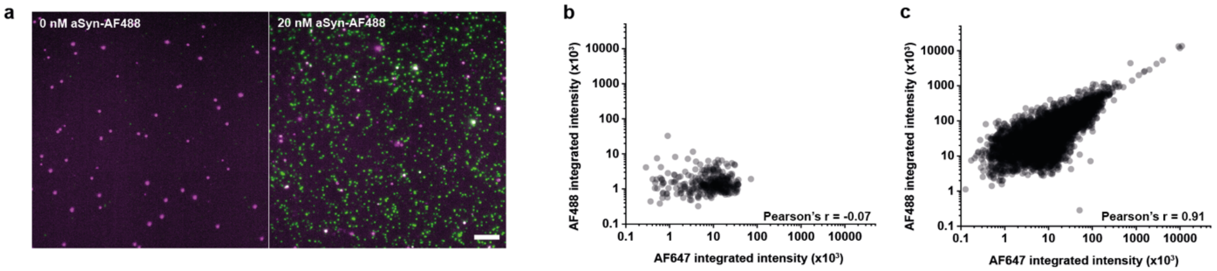

Fig. S4. Antibody intensity correlates with oligomer size. a Representative TIRFM images of 0 nM or 20 nM surface-captured α-syn-AF488 (green) as indicated, probed with AF647-labelled SYN-CT2 antibody (magenta), scale bar 5 µm. The correlation between antibody-derived signal (AF647) and α-syn-derived signal (AF488) was ascertained by plotting the integrated intensity of each channel against the other for all coincident spots across 64 FOVs, both in the absence (b) and the presence of α-syn-AF488 (c). Pearson's correlation coefficient was used to determine the strength of the correlation in each case, producing a significant correlation when 20 nM α-syn-AF488 is present ($p < 2.2x10^{-16}$).

Fig. S5.

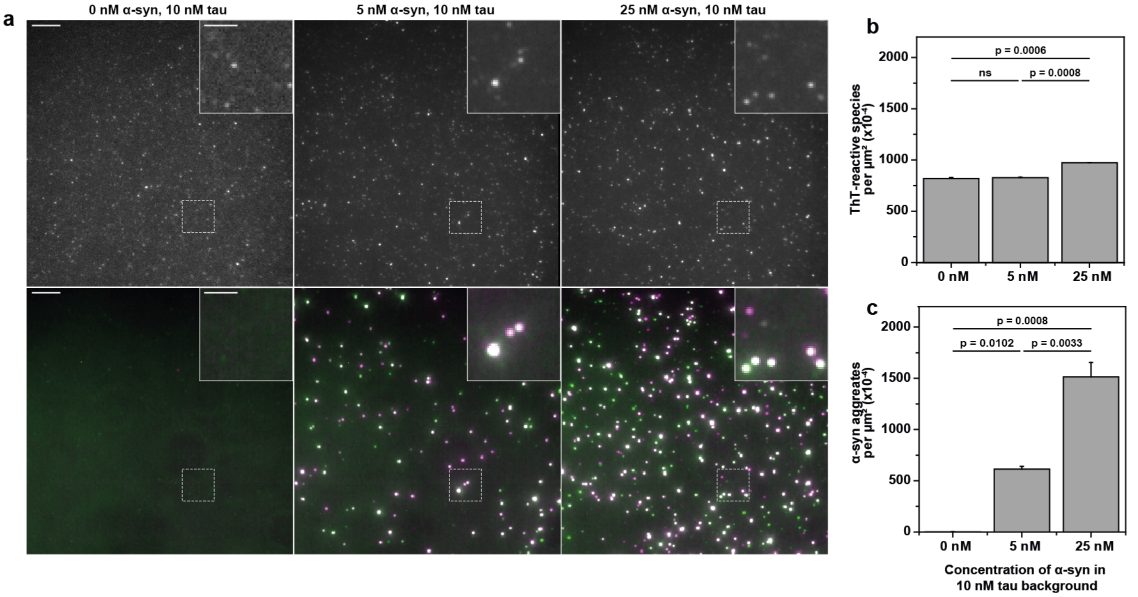

Fig. S5. The STAPull detection antibody confers specificity. a Representative TIRF microscopy images of mixed protein aggregate samples (10 nM tau with 0-25 nM α-syn, as indicated) pulled down by the SYN-CT1 anti-α-syn antibody. Detection used either SAVE ThT imaging to non-specifically detect all amyloid aggregates (top row) or STAPull with anti-α-syn SYN-CT2 to specifically detect α-syn aggregates (bottom row). Scale bars 5 μm in full-frame, 2 μm in inset. **b** Quantification of the number of ThT-reactive species and **c** STAPull coincident events per μm^2 for the dataset presented in a (mean ± SD, n = 2, 64 technical repeats). Statistical analyses carried out by one-way ANOVA, showing significant differences in both groups ($p = 0.00047$ for ThT (b) and $p = 0.00081$ for STAPull (c)), Tukey post-hoc analysis displayed in graph.

Fig. S6.

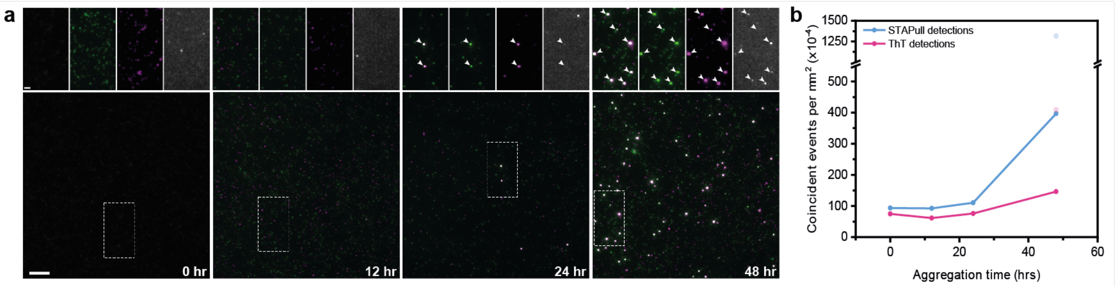

Fig. S6. STAPull detects earlier aggregates than ThT. a Representative STAPull images of α-syn incubated under aggregation-promoting conditions for 0-48 hours, as indicated. Magnified STAPull (left), single-channel (center) and ThT (right) counterstain images of the boxed region are shown above, coincident events representing aggregates are highlighted (white arrowhead) demonstrating a population of STAPull aggregates that are not ThT-reactive. Scale bars 5 μm in full-frame, 1 μm in inset. **b** Quantification of the mean number of aggregates per μm^2 over time as detected by STAPull (blue) or ThT (pink) for the dataset represented in a (mean ± SD, n = 3, 64 FOVs, outliers shown at 48 hours excluded from mean calculations).

Fig. S7.

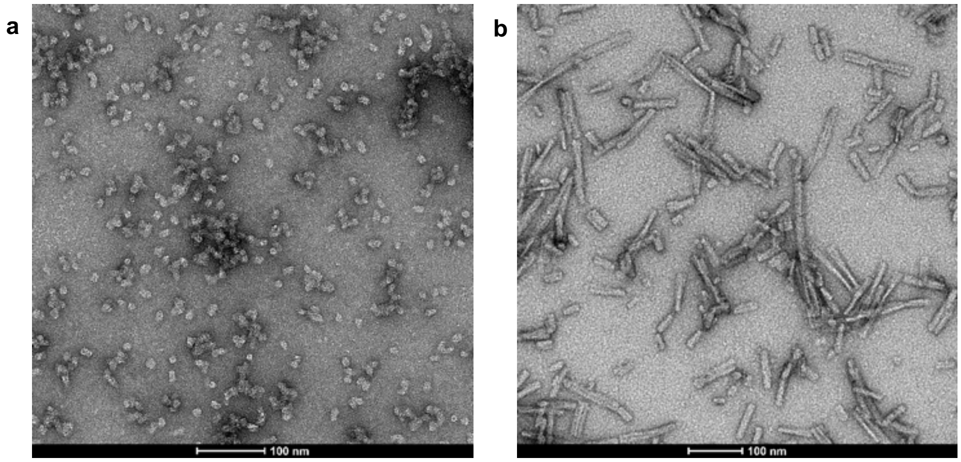

Fig. S7. Structural validation of commercial α-syn constructs using transmission electron microscopy. Representative transmission electron microscopy (TEM) images of commercially sourced recombinant **a** kinetically-trapped α-syn oligomers and **b** pre-formed fibrils. Samples were prepared using the 'direct application method' previously published (*40*). Negative stain TEM images were acquired at 80 Kv on carbon coated 400 mesh copper grids using phosphotungstic acid and uranyl acetate stain.

Fig. S8.

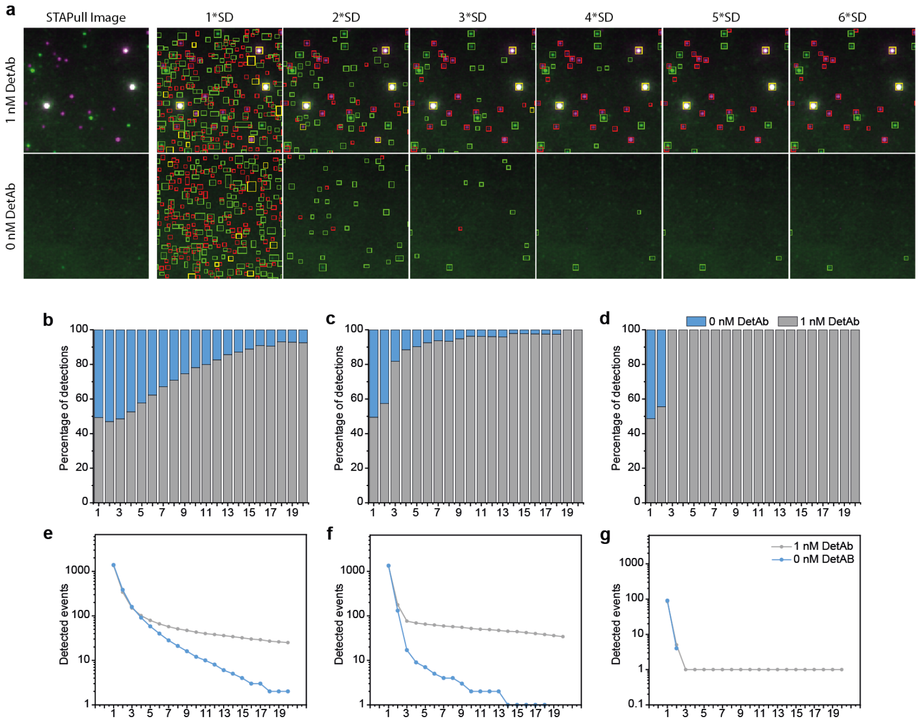

Fig. S8. Empirical determination of the threshold value for ComDet particle detection. a Representative STAPull images of 10 nM α-syn aggregates in the presence (top) or absence (bottom) of 1 nM SYN-CT2 detection antibody, alongside overlaid particle detections obtained for the same image using an intensity threshold set at 1-6 standard deviations above the mean, as indicated, with single channel (green or red boxes) and coincident (yellow boxes) detections shown. **b** The percent of combined detections that are specific to α-syn (grey, based on mean count in the presence of 1 nM detection antibody) and non-specific (blue, based on mean count in the absence of detection antibody) as a function of threshold value for AF488, **c** AF647, and **d** STAPull coincidence. **e-g** The mean particle detections for **e** AF488, **f** AF647, and **g** STAPull coincidence (over 64 technical repeats) in the presence (grey) or absence (blue) of 1 nM detection antibody.

Fig. S9.

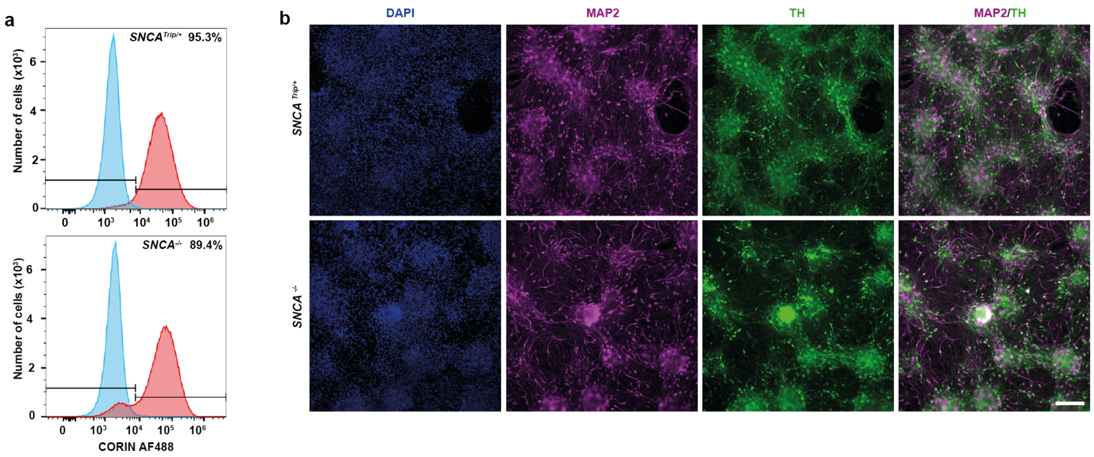

Fig. S9. Differentiation of iPSCs into mDA neurons. a Percentage of day 16 neural progenitor cells expressing cell surface protein CORIN, a floor plate identity marker for *SNCA*$^{Trip/+}$ (top) and *SNCA*$^{-/-}$ (bottom) cultures. **b** Immunostaining of day 98 mDA neurons differentiated from *SNCA*$^{Trip/+}$ (top) and *SNCA*$^{-/-}$ (bottom) iPSCs; DAPI (blue), Tyrosine Hydroxylase (green), Microtubule-associated protein 2 (Magenta). Scale bar is 200 µm in length.

Table S1.

Antibody	Target	Human α-syn monomer k_D [nM]	Human α-syn fibril k_D [pM]	Source	RRID
SYN-NT1	α-syn N-terminal	41.0	463	Gifted by UCB Biopharma	N/A
SYN-CT1	α-syn early C-terminal	0.18	40	Gifted by UCB Biopharma	N/A
SYN-CT2	α-syn late C-terminal	10.0	30	Gifted by UCB Biopharma	N/A
Anti-tau	Tau	Unknown	Unknown	Gifted by UCB Biopharma	N/A
LB509	aSyn 115-122	Unknown	Unknown	Abcam, ab264092	AB_727020
MJFR-14-6-4-2 AF488	Aggregate-specific	KD1: 3290<br>KD2: 0.25	KD1: 0.76 nM,<br>KD2: 0.0028	Abcam, ab216124	AB_2714215
MJFR-14-6-4-2 AF647	Aggregate-specific	KD1: 3290<br>KD2: 0.25	KD1: 0.76 nM,<br>KD2: 0.0028	Abcam, ab216309	AB_2714215

Table S1. Antibodies used in this study. Affinities to monomer and fibrillar α-syn were measured by SPR for SYN-NT1, SYN-CT1 and SYN-CT2. Binding kinetics for MJFR-14 were previously reported by Kumar *et al*. (*41*).

Table S2.

Figure 1	Panel D (Kruskal-Wallis: p = 0.027)		
SiMPull - STAPull	0.54		
SiMPull - Non-treated	0.54		
STAPull - Non-treated	0.02		

Figure 3	Panel B (ANOVA: p = 1.54e-116)	Panel C (ANOVA: p = 1.69e-213)	Panel D (ANOVA: p = 1.65e-167)
α-syn - Aβ	<.00001	<.00001	0.99639
Tau - Aβ	0.62459	0.99991	<.00001
Tau - α-syn	<.00001	<.00001	<.00001
No protein - Aβ	0.4375	0.99757	0.99208
No protein - α-syn	<.00001	<.00001	0.9999
No protein - tau	0.99077	0.99926	<.00001

Figure 4	Panel B (Kruskal-Wallis: p = 2.04e-13)	Panel C (Kruskal-Wallis: p = 5.13e-15)	
Fib - Mon	5.12E-04	1.50E-04	
Fib - Olig	6.31E-14	1.50E-04	
Mon - Olig	3.07E-04	1.49E-15	

Figure 5	Panel B (ANOVA: p = 0.0001)		
Media only - *SNCA-/-*	9.31E-01		
SNCATrip/+ - *SNCA-/-*	2.36E-04		
SNCATrip/+ - Media	1.89E-04		

Table S2. Post-hoc pairwise means comparisons. Dunn's test post-hoc analysis for Kruskal-Wallis statistical tests presented in Fig. 1 and 4, and Tukey post-hoc analysis for one-way ANOVA statistical tests presented in Fig. 3 and 5.

Legend for table S3.

Clinical details of patients and controls along with CSF biomarker analysis.

Legend for data S1

A separate file (.xlsx) for raw data of low sample experiments.

REFERENCES AND NOTES

1. F. Chiti, C. M. Dobson, Protein misfolding, functional amyloid, and human disease. *Annu. Rev. Biochem.* **75**, 333–366 (2006).

2. B. Winner, R. Jappelli, S. K. Maji, P. A. Desplats, L. Boyer, S. Aigner, C. Hetzer, T. Loher, M. Vilar, S. Campioni, C. Tzitzilonis, A. Soragni, S. Jessberger, H. Mira, A. Consiglio, E. Pham, E. Masliah, F. H. Gage, R. Riek, In vivo demonstration that α-synuclein oligomers are toxic. *Proc. Natl. Acad. Sci. U.S.A.* **108**, 4194–4199 (2011).

3. C. A. Lasagna-Reeves, D. L. Castillo-Carranza, U. Sengupta, A. L. Clos, G. R. Jackson, R. Kayed, Tau oligomers impair memory and induce synaptic and mitochondrial dysfunction in wild-type mice. *Mol. Neurodegener.* **6**, 39 (2011).

4. T. Tokuda, M. M. Qureshi, M. T. Ardah, S. Varghese, S. A. S. Shehab, T. Kasai, N. Ishigami, A. Tamaoka, M. Nakagawa, O. M. A. El-Agnaf, Detection of elevated levels of α-synuclein oligomers in CSF from patients with Parkinson disease. *Neurology* **75**, 1766–1772 (2010).

5. L. C. Walker, Proteopathic strains and the heterogeneity of neurodegenerative diseases. *Annu. Rev. Genet.* **50**, 329–346 (2016).

6. M. H. Horrocks, S. F. Lee, S. Gandhi, N. K. Magdalinou, S. W. Chen, M. J. Devine, L. Tosatto, M. Kjaergaard, J. S. Beckwith, H. Zetterberg, M. Iljina, N. Cremades, C. M. Dobson, N. W. Wood, D. Klenerman, Single-molecule imaging of individual amyloid protein aggregates in human biofluids. *ACS Chem. Nerosci.* **7**, 399–406 (2016).

7. M. J. Morten, L. Sirvio, H. Rupawala, E. Mee Hayes, A. Franco, C. Radulescu, L. Ying, S. J. Barnes, A. Muga, Y. Ye, Quantitative super-resolution imaging of pathological aggregates reveals distinct toxicity profiles in different synucleinopathies. *Proc. Natl. Acad. Sci. U.S.A.* **119**, e2205591119 (2022).

8. J.-E. Lee, J. C. Sang, M. Rodrigues, A. R. Carr, M. H. Horrocks, S. De, M. N. Bongiovanni, P. Flagmeier, C. M. Dobson, D. J. Wales, S. F. Lee, D. Klenerman, Mapping surface hydrophobicity of α-synuclein oligomers at the nanoscale. *Nano Lett.* **18**, 7494–7501 (2018).

9. A. Jain, R. Liu, B. Ramani, E. Arauz, Y. Ishitsuka, K. Ragunathan, J. Park, J. Chen, Y. K. Xiang, T. Ha, Probing cellular protein complexes using single-molecule pull-down. *Nature* **473**, 484–488 (2011).

10. G. Je, B. Croop, S. Basu, J. Tang, K. Y. Han, Y.-S. Kim, Endogenous α-synuclein protein analysis from human brain tissues using single-molecule pull-down assay. *Anal. Chem.* **89**, 13044–13048 (2017).

11. A. Chappard, C. Leighton, R. S. Saleeb, K. Jeacock, S. R. Ball, K. Morris, O. Kantelberg, J.-E. Lee, E. Zacco, A. Pastore, M. Sunde, D. J. Clarke, P. Downey, T. Kunath, M. H. Horrocks, Single-molecule two-color coincidence detection of unlabeled α-synuclein aggregates. *Angew. Chem. Int. Ed. Engl.*, **62** e202216771 (2023).

12. L. Tosatto, M. H. Horrocks, A. J. Dear, T. P. J. Knowles, M. Dalla Serra, N. Cremades, C. M. Dobson, D. Klenerman, Single-molecule FRET studies on α-synuclein oligomerization of Parkinson's disease genetically related mutants. *Sci. Rep.* **5**, 16696 (2015).

13. A. A. Hariri, S. S. Newman, S. Tan, D. Mamerow, A. M. Adams, N. Maganzini, B. L. Zhong, M. Eisenstein, A. R. Dunn, H. T. Soh, Improved immunoassay sensitivity and specificity using single-molecule colocalization. *Nat. Commun.* **13**, 5359 (2022).

14. D. A. Armbruster, T. Pry, Limit of blank, limit of detection and limit of quantitation. *Clin. Biochem. Rev.* **29**, S49–S52 (2008).

15. S. W. Chen, S. Drakulic, E. Deas, M. Ouberai, F. A. Aprile, R. Arranz, S. Ness, C. Roodveldt, T. Guilliams, E. J. De-Genst, D. Klenerman, N. W. Wood, T. P. J. Knowles, C. Alfonso, G. Rivas, A. Y. Abramov, J. M. Valpuesta, C. M. Dobson, N. Cremades, Structural characterization of toxic oligomers that are kinetically trapped during α-synuclein fibril formation. *Proc. Natl. Acad. Sci.* **112**, E1994–E2003 (2015).

16. I. van Steenoven, N. K. Majbour, N. N. Vaikath, H. W. Berendse, W. M. van der Flier, W. D. J. van de Berg, C. E. Teunissen, A. W. Lemstra, O. M. A. El-Agnaf, α-Synuclein species as potential cerebrospinal fluid biomarkers for dementia with lewy bodies. *Mov. Disord.* **33**, 1724–1733 (2018).

17. N. K. Majbour, N. N. Vaikath, K. D. van Dijk, M. T. Ardah, S. Varghese, L. B. Vesterager, L. P. Montezinho, S. Poole, B. Safieh-Garabedian, T. Tokuda, C. E. Teunissen, H. W. Berendse, W. D. J. van de Berg, O. M. A. El-Agnaf, Oligomeric and phosphorylated α-synuclein as potential CSF biomarkers for Parkinson's disease. *Mol. Neurodegener.* **11**, 7 (2016).

18. M. Biancalana, S. Koide, Molecular mechanism of Thioflavin-T binding to amyloid fibrils. *Biochim. Biophys. Acta* **1804**, 1405–1412 (2010).

19. N. Lorenzen, S. B. Nielsen, A. K. Buell, J. D. Kaspersen, P. Arosio, B. S. Vad, W. Paslawski, G. Christiansen, Z. Valnickova-Hansen, M. Andreasen, J. J. Enghild, J. S. Pedersen, C. M. Dobson, T. P. J. Knowles, D. E. Otzen, The role of stable α-synuclein oligomers in the molecular events underlying amyloid formation. *J. Am. Chem. Soc.* **136**, 3859–3868 (2014).

20. A. B. Singleton, M. Farrer, J. Johnson, A. Singleton, S. Hague, J. Kachergus, M. Hulihan, T. Peuralinna, A. Dutra, R. Nussbaum, S. Lincoln, A. Crawley, M. Hanson, D. Maraganore, C. Adler, M. R. Cookson, M. Muenter, M. Baptista, D. Miller, J. Blancato, J. Hardy, K. Gwinn-Hardy, α-Synuclein locus triplication causes Parkinson's disease. *Science* **302**, 841 (2003).

21. M. Iljina, G. A. Garcia, M. H. Horrocks, L. Tosatto, M. L. Choi, K. A. Ganzinger, A. Y. Abramov, S. Gandhi, N. W. Wood, N. Cremades, C. M. Dobson, T. P. J. Knowles, D. Klenerman, Kinetic model of the aggregation of α-synuclein provides insights into prion-like spreading. *Proc. Natl. Acad. Sci. U.S.A.* **113**, E1206–E1215 (2016).

22. D. R. Whiten, Y. Zuo, L. Calo, M. L. Choi, S. De, P. Flagmeier, D. C. Wirthensohn, F. Kundel, R. T. Ranasinghe, S. E. Sanchez, D. Athauda, S. F. Lee, C. M. Dobson, S. Gandhi, M. G. Spillantini, D. Klenerman, M. H. Horrocks, Nanoscopic characterisation of individual endogenous protein aggregates in human neuronal cells. *Chembiochem* **19**, 2033–2038 (2018).

23. N. K. Magdalinou, R. W. Paterson, J. M. Schott, N. C. Fox, C. Mummery, K. Blennow, K. Bhatia, H. R. Morris, P. Giunti, T. T. Warner, R. de Silva, A. J. Lees, H. Zetterberg, A panel of nine cerebrospinal fluid biomarkers may identify patients with atypical parkinsonian syndromes. *J. Neurol. Neurosurg. Psychiatry* **86**, 1240–1247 (2015).

24. Z. A. Sorrentino, B. I. Giasson, The emerging role of α-synuclein truncation in aggregation and disease. *J. Biol. Chem.* **295**, 10224–10244 (2020).

25. L. Gaetani, K. Blennow, P. Calabresi, M. Di Filippo, L. Parnetti, H. Zetterberg, Neurofilament light chain as a biomarker in neurological disorders. *J. Neurol. Neurosurg. Psychiatry* **90**, 870–881 (2019).

26. D. Athauda, T. Foltynie, Challenges in detecting disease modification in Parkinson's disease clinical trials. *Parkinsonism Relat. Disord.* **32**, 1–11 (2016).

27. L. Bousset, L. Pieri, G. Ruiz-Arlandis, J. Gath, P. H. Jensen, B. Habenstein, K. Madiona, V. Olieric, A. Böckmann, B. H. Meier, R. Melki, Structural and functional characterization of two α-synuclein strains. *Nat. Commun.* **4** 2575 (2013).

28. P. Liu, M. N. Reed, L. A. Kotilinek, M. K. O. Grant, C. L. Forster, W. Qiang, S. L. Shapiro, J. H. Reichl, A. C. A. Chiang, J. L. Jankowsky, C. M. Wilmot, J. P. Cleary, K. R. Zahs, K. H. Ashe, Quaternary structure defines a large class of amyloid-β oligomers neutralized by sequestration. *Cell Rep.* **11**, 1760–1771 (2015).

29. Z. Krejciova, J. Alibhai, C. Zhao, R. Krencik, N. M. Rzechorzek, E. M. Ullian, J. Manson, J. W. Ironside, M. W. Head, S. Chandran, Human stem cell-derived astrocytes replicate human prions in a *PRNP* genotype-dependent manner. *J. Exp. Med.* **214**, 3481–3495 (2017).

30. K. E. Paleologou, A. W. Schmid, C. C. Rospigliosi, H.-Y. Kim, G. R. Lamberto, R. A. Fredenburg, P. T. Lansbury Jr, C. O. Fernandez, D. Eliezer, M. Zweckstetter, H. A. Lashuel, Phosphorylation at Ser-129 but not the phosphomimics S129E/D inhibits the fibrillation of α-synuclein. *J. Biol. Chem.* **283**, 16895–16905 (2008).

31. W. Hoyer, T. Antony, D. Cherny, G. Heim, T. M. Jovin, V. Subramaniam, Dependence of α-synuclein aggregate morphology on solution conditions. *J. Mol. Biol.* **322**, 383–393 (2002).

32. N. Cremades, S. I. A. Cohen, E. Deas, A. Y. Abramov, A. Y. Chen, A. Orte, M. Sandal, R. W. Clarke, P. Dunne, F. A. Aprile, C. W. Bertoncini, N. W. Wood, T. P. J. Knowles, C. M. Dobson, D.

Klenerman, Direct observation of the interconversion of normal and toxic forms of α-synuclein. *Cell* **149**, 1048–1059 (2012).

33. B. S. Reddy, B. N. Chatterji, An FFT-based technique for translation, rotation, and scale-invariant image registration. *IEEE Trans. Image Process.* **5**, 1266–1271 (1996).

34. M. J. Devine, M. Ryten, P. Vodicka, A. J. Thomson, T. Burdon, H. Houlden, F. Cavaleri, M. Nagano, N. J. Drummond, J.-W. Taanman, A. H. Schapira, K. Gwinn, J. Hardy, P. A. Lewis, T. Kunath, Parkinson's disease induced pluripotent stem cells with triplication of the α-synuclein locus. *Nat. Commun.* **2**, 440 (2011).

35. Y. Chen, K. S. Dolt, M. Kriek, T. Baker, P. Downey, N. J. Drummond, M. A. Canham, A. Natalwala, S. Rosser, T. Kunath, Engineering synucleinopathy-resistant human dopaminergic neurons by CRISPR-mediated deletion of the *SNCA* gene. *Eur. J. Neurosci.* **49**, 510–524 (2019).

36. G. McKhann, D. Drachman, M. Folstein, R. Katzman, D. Price, E. M. Stadlan, Clinical diagnosis of Alzheimer's disease: Report of the NINCDS-ADRDA Work Group under the auspices of Department of Health and Human Services Task Force on Alzheimer's disease. *Neurology* **34**, 939–944 (1984).

37. National Collaborating Centre for Chronic Conditions (UK), *Parkinson's Disease: National Clinical Guideline for Diagnosis and Management in Primary and Secondary Care* (Royal College of Physicians).

38. M. H. Horrocks, L. Tosatto, A. J. Dear, G. A. Garcia, M. Iljina, N. Cremades, M. Dalla Serra, T. P. J. Knowles, C. M. Dobson, D. Klenerman, Fast flow microfluidics and single-molecule fluorescence for the rapid characterization of α-synuclein oligomers. *Anal. Chem.* **87**, 8818–8826 (2015).

39. M. L. Choi, A. Chappard, B. P. Singh, C. Maclachlan, M. Rodrigues, E. I. Fedotova, A. V. Berezhnov, S. De, C. J. Peddie, D. Athauda, G. S. Virdi, W. Zhang, J. R. Evans, A. I. Wernick, Z. S. Zanjani, P. R. Angelova, N. Esteras, A. Y. Vinokurov, K. Morris, K. Jeacock, L. Tosatto, D. Little, P. Gissen, D. J. Clarke, T. Kunath, L. Collinson, D. Klenerman, A. Y. Abramov, M. H. Horrocks, S.

Gandhi, Pathological structural conversion of α-synuclein at the mitochondria induces neuronal toxicity. *Nat. Neurosci.* **25**, 1134–1148 (2022).

40. F. W. Doane, N. Anderson, *Diagnostic Virology—A Practical Guide And Atlas* (Cambridge Univ. Press, 1997).

41. S. T. Kumar, S. Jagannath, C. Francois, H. Vanderstichele, E. Stoops, H. A. Lashuel, How specific are the conformation-specific α-synuclein antibodies? Characterization and validation of 16 α-synuclein conformation-specific antibodies using well-characterized preparations of α-synuclein monomers, fibrils and oligomers with distinct structures and morphology. *Neurobiol. Dis.* **146**, 105086 (2020).
